# Compensating for Language Deficits in Amnesia II: H.M.’s Spared *versus* Impaired Encoding Categories

**DOI:** 10.3390/brainsci3020415

**Published:** 2013-03-27

**Authors:** Donald G. MacKay, Laura W. Johnson, Chris Hadley

**Affiliations:** Psychology Department, University of California, Los Angeles, CA 90095, USA; E-Mails: laurajohnson@ucla.edu (L.W.J.); cdhadley@gmail.com (C.H.)

**Keywords:** amnesic H.M., encoding *versus* retrieval errors, sentence planning, spared encoding categories, language deficits in amnesia, compensation strategies in amnesia

## Abstract

Although amnesic H.M. typically could not recall where or when he met someone, he could recall their topics of conversation after long interference-filled delays, suggesting impaired encoding for some categories of novel events but not others. Similarly, H.M. successfully encoded into internal representations (sentence plans) some novel linguistic structures but not others in the present language production studies. For example, on the Test of Language Competence (TLC), H.M. produced uncorrected errors when encoding a wide range of novel linguistic structures, e.g., violating reliably more gender constraints than memory-normal controls when encoding referent-noun, pronoun-antecedent, and referent-pronoun anaphora, as when he erroneously and without correction used the gender-inappropriate pronoun “her” to refer to a man. In contrast, H.M. never violated corresponding referent-gender constraints for proper names, suggesting that his mechanisms for encoding proper name gender-agreement were intact. However, H.M. produced no more dysfluencies, off-topic comments, false starts, neologisms, or word and phonological sequencing errors than controls on the TLC. Present results suggest that: (a) frontal mechanisms for *retrieving* and *sequencing* word, phrase, and phonological categories are intact in H.M., unlike in category-specific aphasia; (b) *encoding* mechanisms in the hippocampal region are category-specific rather than item-specific, applying to, e.g., proper names rather than words; (c) H.M.’s category-specific mechanisms for *encoding* referents into words, phrases, and propositions are impaired, with the exception of referent gender, person, and number for encoding proper names; and (d) H.M. overuses his intact proper name encoding mechanisms to compensate for his impaired mechanisms for encoding other functionally equivalent linguistic information.

## 1. Introduction

“There are, behind the expressed sequences of behavior, a multiplicity of integrative processes which can only be inferred from the final results of their activity” (Lashley [1], p. 115). This quote outlines the framework, research strategy, and primary focus of this article and its companion, MacKay, Johnson, Fazel, and James [2]. MacKay *et al*. analyzed spoken and written “final results” from amnesic H.M. to infer that (a) his category-specific mechanisms for retrieving words and noun phrases (NPs) are intact (unlike category-specific aphasics’), and (b) he can use his intact retrieval mechanisms to compensate for his impairments in *encoding* novel phrases and propositions [3].

The present research analyzed another type of “final result” (speech errors) to demonstrate that: (a) H.M.’s mechanisms for encoding many types of novel phrases are impaired; (b) but he can encode pictures of unfamiliar people into proper names of the appropriate gender, number, and person; and (c) he can use his intact mechanisms for encoding proper names to compensate for his impaired ability to encode other functionally equivalent linguistic structures for referring to people. Although language represents a cutting edge topic in current research on amnesia (see e.g., [4]), no other studies have examined strategies used by amnesics to compensate for sentence production errors. 

### 1.1. Language, Amnesia, and the Potential of Lashley’s Strategy

To illustrate (a) the usefulness of Lashley’s strategy for providing insights into amnesia, and (b) some background questions that motivated the present research, consider the following excerpt from H.M.’s conversational speech at age 44 in the 182-page transcript of Marslen-Wilson [5]. To illustrate these background questions, we have divided this brief excerpt into four segments.

(1).
  Marslen-Wilson (M-W.): Do you know anything about a war in Vietnam?
(1.1).
  H.M.: … In a way I don’t … know the … anything about it in a way … but … uh … Americans … went over to help … fight over there.
  M-W.: When was that?
(1.2).
  H.M.: In … the date I cannot give.

Segment (1) illustrates what H.M. did and did not know about the Vietnam War in 1970 (17 years after his 1953 lesion): He knew that “Americans went over to help fight” in Vietnam (see (1.1)) but did not know when the Vietnam war began (see (1.2)), and the question is why. Under one explanation, amnesics can only learn novel post-lesion information that is massively repeated (see e.g., [6,7,8,9,10,11,12,13,14,15,16,17,18,19]), so that H.M. knew that Americans fought in Vietnam because this information was massively repeated in his 1965–1970 television viewing, but he did not know that the Vietnam war began in 1965 because this was rarely encountered information in 1970. However, the present application of Lashley’s strategy to H.M.’s speech will call for refinement of this massive repetition principle (see also [2]).

(2).
  M-W.: Yes … went over to fight where? … in Vietnam?
  H.M.: In Vietniam (sic) … was the … and … I think of ... uh … the … uh people that … uh ... are … to free the people that are there that have been held down themselves … by a … in a … governmental things too … the people can’t say or buy or even do what they want to do … they have to do just … what the person says.

Segment (2) continues from where segment (1) left off and illustrates some additional background questions that motivated the present research. Note in (2) the vague, incoherent, ungrammatical, and difficult-to-understand phrases, e.g., “governmental things”, and propositions, e.g., “the people can’t say or buy ... what they want to do” (*what people want to do* is ungrammatical as the object of “say or buy”). H.M. has produced similarly vague, incoherent, ungrammatical, and difficult-to-understand utterances reliably more often than closely matched memory-normal controls in a wide variety of tasks from 1970 to 1999, including experimental tasks (see [12,13,20,21]), spontaneous speech [22], and standardized tests [11]. Like excerpt (2), these data raise two questions: What is the relation between H.M.’s impaired communication and his brain damage? And can H.M. use other, intact brain areas to offset his language impairments, at least in part? To address these questions, the present research will analyze large numbers of H.M.’s vague, incoherent, ungrammatical, and difficult-to-understand utterances in relation to his brain damage.

(3).
  M-W.: Which person says?
(3.1).
  H.M.: … and … I think of **Shek** right off …
  M-W.: Shek?
  H.M.: **Chiang Kai Shek**.
  M-W.: Chiang Kai Shek.
  H.M.: That’s right … **Chiang Kai Shek**.
  M-W.: You think the Americans are fighting against him in Vietnam?
(3.2).
  H.M.: … and … uh … Vietnam is … uh … not ... uh … part of … uh … well it’s … in Asia but not part of China.
  M-W.: No, that’s right …
  H.M.: And … uh … I believe he ... uh ... uh ... I believe the Americans are fighting against the Soviet Union …
  M-W.: Where?
(3.3).
  H.M.: In **Chiang Kai Shek** … uh … not **Chiang Kai Shek** but the … uh ... well … Vietnam.

Segment (3) continues from where segment (2) left off and contains two highlighted speech errors that raise further questions. In (3.2), H.M. indicated awareness that he had substituted one proper name (*Chiang Kai Shek*, the Chinese dictator) for another (*Ho Chi Minh*, the Vietnamese communist leader) in (3.1). This perfectly normal error + error detection sequence is noteworthy because H.M. detects other types of self-produced errors reliably less often than memory-normal controls in a wide variety of tasks (for a review, see [23]). 

Similarly in (3.3), H.M. substituted one proper name (*Chiang Kai Shek*) for another (*Vietnam*), followed by (a) “uh” and “not” (error markers indicating that an error has occurred), and (b) an error correction. This perfectly normal sequence (error + error marker(s) + correction) is also noteworthy because H.M. reliably more often than memory-normal controls (a) fails to produce error markers to signal occurrence of self-produced errors involving a wide range of other word types, and (b) fails to correct those errors (see [24])*.*

Such examples raised three questions addressed in the present research: Why does H.M. detect, mark, and correct proper name errors, but not other types of errors? Are proper names somehow immune to H.M.’s communication deficits involving other word types? And if so, does H.M. use proper names to overcome or compensate for his other linguistic impairments? 

To answer these questions, we applied Lashley’s [1] strategy to H.M.’s use of proper names and other functionally equivalent linguistic structures on a standardized language production test, with special attention to speech errors. Because theories of the mechanisms underlying normal speech production must explain the regularities in how production breaks down into errors (see [1]), we hoped to discover regularities in H.M.’s speech errors that carried implications for the neural mechanisms underlying normal sentence production, and consistent with that hope, our results called for refinement of current theories of the binding processes underlying everyday sentence planning. 

### 1.2. Structure of the Present Paper

The present research consists of two studies. The question in Study 1 was: Can the proposition-level compensation hypothesis of MacKay *et al.* [2] be extended to words and phrases? Under the proposition-level hypothesis, H.M. retrieved preformed propositions via free association on the Test of Language Competence (TLC; [25]) and used coordinating conjunction *and* to conjoin them, thereby satisfying the TLC instruction to produce “a single grammatical sentence” because any propositions conjoined via *and* form a grammatical (but not necessarily accurate, coherent, or relevant) sentence. This strategy served to compensate for H.M.’s inability to construct novel sentence-level plans but yielded overuse of *and* relative to memory-normal controls (who never used *and* to conjoin propositions generated via free association).

Under the analogous Study 1 hypothesis, H.M. will retrieve familiar words and phrases via free association on the TLC to compensate for his inability to encode novel phrase-level plans. Because no previous study has compared word- and phrase-level free associations for H.M. *versus* memory-normal controls on the TLC, testing this hypothesis was important for addressing the more complex compensation processes examined in Study 2. 

Study 2 conducted detailed analyses of six overlapping categories of speech errors produced by H.M. and memory-normal controls on the TLC: major *versus* minor errors, retrieval *versus* encoding errors, and commission- *versus* omission-type encoding errors. By definition, minor errors do not disrupt ongoing communication because they are corrected (with or without help from a listener). However, major errors disrupt communication because (a) they are uncorrected with or without prompts from a listener (see [24]), and (b) they reduce the grammaticality, coherence, comprehensibility, or accuracy of an utterance (see [24]). Example (4) illustrates a minor (corrected) error, and examples (5a–d) illustrate (hypothetical) major errors [26]. For example, “In the they got sick” instead of *in the interim they got sick* in (5a) is a major error because it is ungrammatical, uncorrected, and disrupts communication.

(4). *Put it on the chair*.→“Put it on the table … I mean, chair.” (minor error)
(5a). *In the interim they got sick*.→“In the they got sick.” (uncorrected major error)
(5b). *I want either* *some cake or that pie*.→“I want either some cake but some pie.” (uncorrected major error)
(5c). *I* *want either* *some cake or that pie*.→“I want either some or that pie.” (uncorrected major error)
(5d). *She eats cake*.→“She exists cake.” (uncorrected major error)

In minor retrieval errors, speakers substitute an unintended unit (e.g., phrase, word, or speech sound) for an intended unit in the same category (e.g., NP, noun, or vowel), consistent with the sequential class regularity (see [2]). For example, (6) is a phrase-level retrieval error because the speaker retrieved one NP (*our laboratory*) instead of the another (*a computer*); (7) is a word-level retrieval error because the speaker retrieved one preposition instead of another; and (8) is a phonological retrieval error because the speaker retrieved one initial consonant instead of another (examples from [27]).

(6). *We have a computer in our laboratory*.→“We have our laboratory in …” (minor phrase retrieval error)
(7). *Are you going to be in town on June 22nd*?→“Are you going to be on town …” (minor word retrieval error)
(8). *a reading list*→“a leading list” (minor phonological retrieval error)

By contrast, encoding errors occur when speakers either (a) fail to conjoin units that should be conjoined in an intended utterance or sentence plan (omission-type encoding errors), or (b) conjoin units that should not be conjoined (commission-type encoding errors). When uncorrected despite prompts, all encoding errors are major. For example, (5b) is a major commission-type encoding error because it was uncorrected, and the hypothetical speaker conjoined *either* with *but* rather than *or*; and (5c) is a major omission-type encoding error because it was uncorrected, and the hypothetical speaker failed to conjoin the determiner *some* with the noun *cake* to form the NP *some cake*. Defined with greater precision and generality, encoding errors violate conjunction constraints, also known as selection restrictions or agreement rules, which specify the necessary and permissible relations between the words in phrases, phrases in propositions, and propositions in sentences. 

To anticipate the main results of Study 2, H.M. produced no more minor retrieval errors than memory-normal controls on the TLC, but he produced reliably more major encoding errors (both omission-type and commission-type) than the controls for many but not all categories of linguistic-referential units. In particular, H.M. produced reliably more major encoding errors than controls when conjoining referent gender with pronouns, e.g., referring to a man as “she” or to a woman as “he”, but he produced no major encoding errors when conjoining referent gender with proper names, e.g., correctly referring to a man as “David” or a woman as “Melanie”. Based on these and other findings, we will conclude that H.M. overused proper names relative to memory-normal controls in MacKay *et al.* [2] because using proper names offset his inability to encode other functionally equivalent structures. 

### 1.3. Studies 1–2: Participants, Database and Shared Procedures

The participants were amnesic H.M. and eight healthy, memory-normal controls carefully matched for highest educational degree (high school), native language (English), background (semi-skilled labor), age at time of test (approximately 72 years), and mean verbal and performance IQ scores (approximately 112). MacKay *et al.* [2] review H.M.’s bilateral damage to the hippocampal region caused by his 1953 sub-orbital suction surgery, his bilateral cerebellar damage due to long-term dilantin use later in life, and the cortical and vascular changes observed in Salat *et al.* [28]. However, cerebellar involvement in the present results is unlikely, and H.M.’s cortical and vascular changes almost certainly followed the present (1998) studies (see [23]). 

The supplementary materials provide the database, which was identical in Studies 1–2: the full set of transcribed responses (3720 words) of H.M. and eight memory-normal controls on the TLC administered in MacKay *et al.* [11]. The goal on each of the 150 (overall) trials was to create a single grammatical sentence that accurately described a picture and contained two or three target words typed below it (see [11] for further details, including transcription procedures). 

Analyses of word- and phrase-level free associations in Study 1 required the development and implementation of procedures for classifying word- and phrase-level free associations, and analyzing the six classes of errors in Study 2 (major *versus* minor, retrieval *versus* encoding, and omission- *versus* commission-type encoding errors) required the development and implementation of procedures for specifying speaker intent. However, the units of analysis (words and phrases) were identical in both studies, as were the statistical conventions: Meaningful statistical comparisons required raw score differences equal or greater than 4.0 for H.M. *versus* the control mean (as in sign tests, where the minimum signed difference for statistical reliability is 0 *versus* 4); the upper limit difference between H.M. and the control mean was 6.0 *SD*s when the standard deviation (*SD*) for the control mean was 0.0; and differences between H.M. and the control mean had to equal or exceed 2.0 *SD*s to be considered reliable (see the supplementary materials for detailed justifications of these non-arbitrary conventions). 

## 2. Study 1: Word- and Phrase-Level Free Association: A Compensation Strategy

To evaluate and possibly extend the compensation hypothesis developed in MacKay *et al.* [2], Study 1 examined (a) whether H.M. produces more word- and phrase-level free associations than controls on the TLC, and (b) whether his free associations serve to compensate for his inability to construct phrase-level plans that are novel, coherent, accurate, and grammatical. No other study has examined word- and phrase-level free associations on the TLC or how they might offset H.M.’s language production deficits. 

As classically defined (see [29]), free associations express thoughts that are inappropriate or unrelated to the current situational or conceptual context, but strongly related to information in immediate or long term memory. Consistent with this definition, word- and phrase-level free associations occur when speakers produce a word or familiar phrase that is unrelated in meaning to its situational, conceptual, or utterance context but strongly related to information in immediate or long term memory. For example, the comment *that’s in her way* is a phrase-level free association when discussing how *she wants things done her way* because (a) the word *way* has fundamentally different meanings in *that’s in her* way and *she wants things done her way*, and (b) the familiar verb phrases *wants things done her way* and *is in her way* are connected in long term memory via the shared phonological form *way*. Because H.M. produced reliably more proposition-level free associations than memory-normal controls on the TLC (see [2]), we expected him to produce reliably more word- and phrase-level free associations in Study 1, and the question was whether and how his word- and phrase-level free associations could have compensated for his problems in creating sentences that are novel, coherent, accurate, and grammatical on the TLC.

### 2.1. Methods

To score word- and phrase-level free associations in the TLC database, three judges (not blind to H.M.’s identity) received: (a) the word-picture stimuli in MacKay *et al.* [11]; (b) the transcribed responses to each word-picture stimulus; (c) a definition of phrase-level free associations (two or more words in an utterance that were closely related to each other but unrelated or inappropriate to their situational and/or within-utterance context); and (d) hypothetical examples of word- and phrase-level free associations unrelated to the TLC transcripts. The judges then marked word- and phrase-level free associations on their transcripts, and examples confirmed by two or more judges were marked in a final transcript. A single judge then coded each word- and phrase-level free association in the final transcript as having direct, indirect, or no possible benefit to TLC performance. Direct benefit was scored when free associations helped render an utterance grammatical or incorporated one or more of the TLC target words. Indirect benefit was scored when word- or phrase-level free associations seemed to facilitate TLC performance in some other way. 

### 2.2. Results

#### 2.2.1. Main Results

Table 1 provides the final list of word- and phrase-level free associations on the TLC, labeled (9)–(21) to facilitate discussion of their benefits to TLC performance. H.M. produced 14 word- and phrase-level free associations *versus* a mean of 0.0 (*SD* = 0) for the memory-normal controls, a reliable 6.0 *SD* difference by convention.

**Table 1:** Word- and phrase-level free associations in the TLC transcripts, with descriptions in parentheses and type of benefit in brackets.

(9). H.M.: “Before at first you cross across.” (free association 1: *at*–*first*: association from the target word *first* to the phrase *at first*; free association 2: *across*–*cross:* associationfrom the target word *across* to the phonologically similar *cross*) [direct benefit]
(10). H.M.: “Since they’ve got their coffee already he isn’t—they just want their uh pie and the piece of this pie up here because the cake is down here.” (*pie–cake*: free association from the target word *pie* to the semantically similar *cake*) [indirect benefit]
(11). H.M.: (in response to the question “Do you know what the word *either* means?”): “Or.” (*either–or*: free association) [indirect benefit]
(12). H.M.: “Well he’s putting the price of it and price of thing.” (*it–thing*: free association; see text for discussion) [indirect benefit]
(13). H.M.: “price of thing what it is...” (*thing*–*what it is*: free association; see text for discussion) [indirect benefit]
(14). H.M.: “and he’s waitin’ to be waited on.” (*waitin’–waited on*: free association) [indirect benefit]
(15). H.M.: “I like some her … what she had.” (*her–she*: free association) [indirect benefit]
(16). H.M.: “and uh coffee is in there because heat a solid...” (*liquid–solid*: free association) [indirect benefit]
(17). H.M.: “and this is not liquid but only ice.” (*liquid–not liquid*: free association) [indirect benefit]
(18). H.M.: “A driving wanna drive some place and this bus is stopped up there.” (*driving–drive*: free association) [indirect benefit]
(19). H.M.: “David wanted him to fall and to see what lady’s using to pull himself up besides his hands.” (*to fall* and *to see*: free associations to the concept *what David might have wanted*; see text for explanation) [indirect benefit]
(20). H.M.: “Because it’s wrong for her to be and he’s dressed just as this that he’s dressed and the same way—as her.” (*as* *her–as him*: free association; see text for explanation) [indirect benefit]
(21). H.M.: “I want some of that pie either some pie and I’ll have some.” (*either* *want some*–*have some*: free association) [direct benefit]

#### 2.2.2. Subsidiary Results

Three of H.M.’s word- and phrase-level free associations were scored as having direct benefit, 11 as having indirect benefit, and 0 as having no possible benefit to his TLC performance (see Table 1). Two direct benefit examples appear in (9), H.M.’s initial response to three target words (*before*, *first*, *across*) heading a picture of a father and two young children at a sidewalk intersection looking at a traffic light that reads, “Don’t walk”. H.M.’s response, “Before at first you cross across” began with the first target word, *before*, followed by *at first*, a familiar phrase ostensibly formed via free association with *first*, the second target word. H.M. then retrieved the verb *cross*, ostensibly via free association with the phonologically similar target word *across*, and finally, *cross* triggered the familiar phrase *you cross*, again via free association. Finally, H.M. concatenated all of these retrieved forms into (9), an ungrammatical, incoherent, and difficult to comprehend response that nonetheless included all three target words (in italics): “*Before* at *first* you cross *across*.” By hypothesis, H.M.’s *across*–*cross*, *at*–*first*, and *you*–*cross* free associations therefore directly benefited his TLC performance in two ways. First, these free associations incorporated all three target words, a direct benefit with respect to the TLC instructions. Second, H.M. could retrieve the familiar phrases *cross*–*across* and *at*–*first* via his intact retrieval mechanisms, a direct benefit given his inability to create novel phrase- and sentence-level plans. 

Examples scored as having indirect benefits to TLC performance appear in (12) and (13). In (12), H.M. first produced “the price of it”, then “price of thing” ostensibly via free association with *it*, followed by “what it is”, a free associative definition of *thing* (see (13)). As one possible indirect benefit, H.M.’s *it*–*thing* and *thing*–*what it is* (although redundant and free associative) satisfied a general demand characteristic common to all communication: to produce an easily comprehended utterance (from H.M.’s perspective), thereby compensating for his lesion-induced inability to form novel sentence plans that are coherent, grammatical, and easily understood. As another possible indirect benefit, these free associations satisfied the most basic demand characteristic of the TLC: to say *something* about the TLC picture (regardless of how incomplete, redundant, incoherent, tangential, or ungrammatical). 

### 2.3. Discussion

Word- and phrase-level free associations are rare in normal everyday speech production. For example, no studies of normal speech errors have reported free associations resembling “Before at first you cross across” (see e.g., [30]). However, neither experimental procedures *per se* nor the present TLC task were responsible for H.M.’s word- and phrase-level free associations: H.M. has produced reliably more word- and phrase-level free associations than carefully matched memory-normal controls in a wide range of tasks, including non-experimental tasks such as conversational speech (see Table 2 for examples of H.M.’s word- and phrase-level free associations in [5,12,13,22,24]). Nor were H.M.’s word- and phrase-level free associations attributable to his age when tested on the TLC: H.M. has produced qualitatively similar word- and phrase-level free associations (and reliably more than age-matched memory-normal controls) from age 44–47 to age 71–72.5 (compare Table 1, Table 2). 

**Table 2:** H.M.’s word- and phrase-level free associations in five earlier tasks with explanations, typical examples, and age at test.

Task	H.M.’s Age at Test	H.M.’s Word- and Phrase-level Free Associations: Typical Examples with Explanations
Answering questions about common childhood experiences	Age 44 (data from [22])	H.M. (answering the question *Do you remember any of the kids there in kindergarten?*): “Uh, just ... uh ... was a private kindergarten, and being on Burnside Avenue.” *Explanation*: Free association from *kindergarten* to the location and type of kindergarten, without answering the question about *the kids in kindergarten*.
Describing two meanings in short ambiguous sentences	Age 47 in [13]	H.M. (describing one of the meanings of the ambiguous sentence, *The marine captain liked his new position*): “That’s why he liked the position too, because he was above them and of all, most of all.” *Explanation*: Irrelevant free association from *of all* to the associated phrase *most of all*.
Describing two meanings of isolated ambiguous words and phrases	Age 72.5 in [12]	H.M. (describing two meanings for the ambiguous word *lots*): “And that could be many or more.” *Explanation*: Irrelevant free association from *many* to *more* (*lots* doesn’t necessarily mean *more*). H.M. (defining the “draw lots” meaning of the word *lots*): “I think it’s, uh, probably, straw … long and short ones.” *Explanation*: A normal definition of this meaning of *lots* might run: *a way of choosing between alternatives by chance*.However, H.M. describes an associate usable for drawing lots without defining *lots*: *long and short pieces of straw*.
Describing captioned cartoons	Age 71 in [24]	H.M. (describing a ghost in a cartoon): “And, uh, I can’t tell just what—she possibly wants to make it her way, only her way. They’re in her way.” *Explanation*: Irrelevant free association from *way* in *her* *way* and *only her way* to “They’re in her way” (note the differing meanings of *way* here).
Answering autobiographical questions in conversational speech	Age 44 in [5]	H.M. (responding to the question *Have you ever heard of anybody called Martin Luther King*?): “Well, in a way that he … well … everything was, I guess … we … er … better explain it … the way … everything was OK for everyone else but … er … just what he’s done, it’s got to be just right … their .. they can do anything, it doesn’t make any difference, but what I do is *right*, that’s .. *it* ..” (W.M-W.: “I’m not … so what was he saying, what was he doing?”) “Well, in a way, he was just … telling the people in a way that no matter they could think of things they wanted to and everything but … er … his way was *the* way.” *Explanation*: Irrelevant free association from four prior uses of *way* to “his way was *the* way”.

Present results therefore replicate MacKay *et al.* [12,13,22,24] and Marslen-Wilson [5] with an important extension: Participants were instructed to produce grammatical sentences on the TLC but not in earlier studies. In short, free associative responses such as “Before at first you cross across” indicate that H.M. has difficulty creating grammatical sentences, even when instructed to do so. This contrasts with the typical control participant, where a coherent and grammatical sentence plan clearly guided responses such as, “First they waited before walking across the street” (for a detailed theoretical account of how normal speakers might construct such a sentence plan, see [22] and [12]). 

By hypothesis, H.M.’s word- and phrase-level free associations on the TLC reflect attempts to compensate for his difficulties in accurately describing a picture using two or three target words in a single grammatical sentence. This compensation assumed two forms: direct facilitation of H.M.’s TLC performance, e.g., by increasing target word inclusion, and indirect facilitation, e.g., by rendering his utterances more easily understood. 

The functions and effects of H.M.’s word-, phrase-, and proposition-level free associations were therefore similar: All three (a) enabled H.M. to use his intact retrieval processes to offset his inability to create readily understood phrases and sentences that were novel, coherent, and grammatical (see also [5,11,13,22,24,31,32]) and (b) had undesirable side effects, as the redundancy in “the price of it and price of thing what it is” illustrates (see also [2]).

## 3. Study 2: Procedures for Analyzing Speech Errors in the TLC Database

### 3.1.Analytic Procedures Shared across Different Types of Speech Errors

To distinguish major *versus* minor and retrieval *versus* encoding errors, we followed a standard speech error definition in use since 1895 (see [1,23,33,34,35,36]): Speech errors are unintended outputs that require correction because they violate a norm that the speaker implicitly or explicitly knows, accepts, and usually follows. 

Consistent with this definition, Study 2 adopted three procedures for excluding non-errors reflecting deliberate obfuscation, ignorance, intentional humor, guessing, and false starts. First, we questioned participants about their anomalous utterances so as to distinguish genuine errors such as (22a) from otherwise similar false starts such as (22b), where the speaker initially intended to say (22c) but shifted to (22d) in simple to communicate something that seemed more desirable at the time.

(22a). *She put the box in the table … I mean, on the table.* (genuine word substitution error followed by a correction)
(22b). *I’d like a* (“ay”) *… an apple.* (false start: “ay” shifted to *an*)
(22c). *I’d like a* (“ay”) *pear.* (initial plan or intended output)
(22d). *I’d like an apple.* (revised plan or intended output)

Second, we ruled out ignorance by ensuring that our participants’ error-free speech generally followed the norm that their anomalous (ungrammatical or difficult-to-understand) utterance(s) violated. Third, as discussed next, we reconstructed speaker intent via “best possible correction” (BPC) procedures that overcome the limitations while maintaining the strengths of three traditional analytic procedures: the ask-the-speaker, speaker-correction, and most-likely-intent procedures. 

#### 3.1.1. The Ask-the-Speaker Procedure

In speech error studies using this procedure, observers ask speakers what they intended to say after they violate the instructions in experimental settings (see e.g., [36]) or violate a familiar rule or constraint in conversational settings (see e.g., [33,37,38,39,40]). As drawbacks, ask-the-speaker procedures require time-consuming interruptions of an ongoing task or conversation, and are useless when speakers (a) deny their errors (as happens with anosognosic aphasics; see [41,42,43]), or (b) are unwilling or unable to state their intentions (as happens with H.M.: Although generally cooperative, H.M. does not state his intentions when asked, even after violating a rule that he usually follows in his conversational speech; see, e.g., [24]). 

#### 3.1.2. The Speaker-Correction Procedure

If someone says, *Put the box in the … I mean*, *on the table*, the intended utterance was clearly *Put the box on the table*, and researchers can often infer intent from how speakers correct their errors. However, this speaker-correction procedure has a major limitation: Many errors remain uncorrected, e.g., about 45% in the case of everyday word substitutions (see [44]). 

#### 3.1.3. The Most-Likely-Intent Procedure

Researchers (e.g., [27,30,34,45]) often use situational or pragmatic context to infer the most likely intent underlying anomalous utterances such as *Put the box in the table in the kitchen* instead of *Put the box on the table in the kitchen*. Although valid and reliable with highly constrained contexts, e.g., the instructions, pictures, and pre-specified target words on the TLC, such most-likely-intent inferences can nonetheless conflate genuine errors with ignorance, intentional humor, dialect differences, and deliberate rule violations in less constrained utterance contexts.

#### 3.1.4. BPC Procedures

Table 3 outlines the BPC procedures adopted in Study 2 for reconstructing the intended utterances of H.M. and the controls on the TLC. As shown in Table 3, BPC procedures incorporate features of ask-the-speaker, speaker-correction, and most-likely-intent procedures, but (a) are applicable to uncorrected errors and speakers unwilling or unable to state their intentions when asked, and (b) do not conflate errors with ignorance, intentional humor, dialect differences, or deliberate rule violations. 

**Table 3:** Criteria and procedures for determining the best possible correction (BPC) for any utterance and any speaker. Adapted from MacKay *et al.* [24].

Criterion 1: The BPC corresponds to a speaker’s stated intention when questioned or in the case of corrected errors, to their correction, whether self-initiated or in response to listener reactions.
Criterion 2: When criterion 1 is inapplicable, judges suggest as many corrections as possible based on the sentence and pragmatic (or picture) context and rank these alternative error corrections via procedures 1–4. Then the ranks are summed and BPC status is assigned to the candidate with the highest summed rank.
Procedure 1: Assign a higher rank to BPC candidates that retain more words and add fewer words to what the participant actually said.
Procedure 2: Assign a higher rank to BPC candidates that better comport with the pragmatic situation (or picture) and the prosody, syntax, and semantics of the speaker’s utterance.
Procedure 3: Assign a higher rank to BPC candidates that are more coherent, grammatical, and readily understood.
Procedure 4: Assign a higher rank to BPC candidates that better comport with the participant’s use of words, prosody, and syntax in prior studies (see [24] for ways to rule out possible hypothesis-linked coding biases using this procedure).

### 3.2. Scoring and Coding Procedures Shared across Different Types of Speech Errors

To score major errors, three judges (not blind to H.M.’s identity) received: (a) the 21 TLC word-picture stimuli; (b) the transcribed responses of H.M. and the controls; (c) a definition of major errors; and (d) typical examples of major errors unrelated to the TLC (e.g., (5a–d)). Using the definition and examples, the judges then marked major errors on the transcribed responses, and an error was scored in a final transcript when two or more judges were in agreement.

We next followed the procedures and criteria in Table 3 to determine the BPC for each response. These BPCs allowed us to score omission-type CC violations (due to omission of one or more concepts or units in a BPC, e.g., *friendly* in *He tried to be more ...*) and commission-type CC violations (due to substitution of one concept or element for another in a BPC, e.g., *himself* substituted for *herself* in *to see what lady’s using to pull himself up*). 

Finally, using Dictionary.com and the sentence context, we coded the syntactic categories of the intended words, phrases, and propositions in the BPCs. *Prepositional phrases* were defined as a preposition plus an NP. *NPs* as a noun plus (optional) determiners, adjectives, modifier, or complements, *verb phrases* (VPs) as a verb plus an (optional) auxiliary verb, adverb, prepositional phrase, complement or object NP (for transitive verbs only), and *propositions* as a pronoun, noun, or NP, plus a VP (following [46,47,48,49]). 

## 4. Study 2A: H.M.’s Use of Proper Names: Another Compensation Strategy

The goal of Study 2A was to understand why H.M. overused proper names relative to memory-normal controls in MacKay *et al.* [2]. Under our working hypothesis, (a) H.M. produces encoding errors involving pronouns (e.g., *she*), common nouns (e.g., *woman*), and NPs with common noun heads (e.g., *this woman*) because his mechanisms for encoding gender, number, and person via these ways of referring to unfamiliar people are impaired, but (b) H.M. produces proper names without encoding errors because his mechanisms for encoding the gender, number, and person of unfamiliar people (or their pictures) via proper names are intact, and (c) H.M. uses his spared encoding mechanisms to compensate for his impaired ones, causing overuse of proper names for referring to people.

This proper name compensation hypothesis raised several questions addressed in Study 2A. One was: Relative to memory-normal controls referring to unfamiliar people in TLC pictures, does H.M. produce reliably more encoding errors involving gender (male *versus* female), number (singular *versus* plural), and person (human *versus* non-human) using pronouns, common nouns, and NPs with common noun heads, indicating impairment of his encoding mechanisms for these ways of referencing people? 

We chose gender, number, and person encoding errors as our dependent measure in Study 2A for reasons related to our working hypothesis. First, conjunction constraints (CCs) governing gender, person, and number apply alike to all four ways of referring to people addressed in our working hypothesis: pronouns, common nouns, common noun NPs, and proper names. Second, encoding errors are uncorrected, ungrammatical errors that violate CCs for conjoining or encoding two or more related categories of concepts. For example, the sentence *She* (*this lady*, *Mary*)*hurt himself* violates the CC that that reflexive pronouns (here, *himself*) must agree in gender with their pronoun, common noun, or proper noun antecedent (here, *she*, *this lady*, or *Mary*), as in *She* (*this lady*, *Mary*) *hurt herself*. Our working assumption that H.M.’s mechanisms for encoding unfamiliar people in TLC pictures are impaired therefore predicted reliably more violations of gender, person, and number CCs for H.M. than controls with completely intact encoding mechanisms. Third, our working assumption that H.M.’s mechanisms for encoding proper names are intact predicted no more violations of gender, person, and number CCs for H.M. than controls using proper names to refer to unfamiliar people in TLC pictures. 

### 4.1. Methods

The participants and database were identical to Study 1. The analytic, scoring, and coding procedures were as discussed earlier.

### 4.2. Results

Study 2A analyses fell into two categories: general analyses (of major *versus* minor errors and omission- *versus* commission-type CC violations) and specific analyses relevant to proper name compensation. 

#### 4.2.1. General Analyses of CC Violations

##### 4.2.1.1. Major *versus* Minor CC Violations

CC violations in the TLC transcripts were always major (uncorrected and ungrammatical) and never minor (corrected and consistent with the syntactic class regularity). The mean number of major CC violations was 2.22 per response for H.M. *versus* 0.13 for the controls (*SD* = 0.20), a reliable 10.43 *SD* difference. 

##### 4.2.1.2. Major Omission-Type CC Violations

Table 4 provides a complete list of major omission-type CC violations for H.M. and the controls in Study 2, classified by the syntactic category of the units involved. H.M. produced 0.50 omission-type CC violations per response, *versus* a mean of 0.01 for the controls (*SD* = 0.10), a reliable 4.90 *SD* difference. 

**Table 4:** Major omission-type violations of conjunction constraints (CCs) involving determiners, nouns, noun phrases (NPs), and verb modifiers in Study 2 organized by type of constraint.

**Major Violations of Determiner-NP Conjunction Constraints**	
	H.M.: “Is it crowded and it just pointed out this bus is up here and it’s crowded school bus.” (BPC: *a* crowded school bus) [H.M. failed to conjoin the determiner *a* to the NP *a crowded school bus*] H.M.: “If they don’t use legs like he does … and his hands, they could fall.” (determiner BPC: use *their* legs) [H.M. failed to conjoin the pronominal determiner *their* to the NP *their legs*] H.M.: “it’s on common street...” (determiner BPC: on *a* common street) [H.M. failed to conjoin the determiner *a* to the NP *a common street*] H.M.: “Well he’s putting the price of it and price of thing what it is…” (determiner BPC: and *the* price of X (*this*?) thing) [H.M. failed to conjoin the determiner *the* to the NP *the price*] H.M.: “David wanted him to fall and to see what lady’s using to pull himself up besides his hands.” (determiner BPC: to see what *this* lady’s using) [H.M. failed to conjoin the demonstrative determiner *this* to the NP *this lady*] Control participant: “Salesman’s talking to…” (BPC: *a* or *the* salesman’s talking to) [This participant failed to conjoin a determiner to the NP *a* or *the* *salesman*] H.M.: “I want some of that pie either some pie and I’ll have some.” (BPC: and I’ll have some X (*cake*?)) [H.M. failed to conjoin the noun *cake* to the determiner *some* in the NP *some* *cake*] Control participant: “Woman’s telling the baker…” (BPC: *a* or *this* woman’s telling...) [This participant failed to conjoin a determiner to the NP *a* or *the* *woman*]
**Major Violations of Verb-NP Conjunction Constraints**	
	H.M.: “Well you—she wants one thing and he wants another thing and the fresh are not—are not. Doesn’t say that, it says not.” (BPC: the fresh *fruit* are not) H.M.: “He had this (inaudible) … no, she’s taking that suit and he wants to take it … and he’s trying to sell.” (BPC: trying to sell *it* or *that suit*) [H.M. failed to conjoin an object to a VP such as *sell it* or *sell that suit*] Control participant: “This person is showing the lady who’s gonna sit.” (BPC: this person is showing *the sign to* the lady who’s gonna sit) [This participant failed to conjoin an object to the VP *is showing* and a preposition to *the lady*]
**Major Violations of Preposition-NP Conjunction Constraints**	
	H.M.: “Before at first you cross across.” (BPC: you cross across X (*the street*?))
**Major Violations of Copular Complement Conjunction Constraints **	
	H.M.: “Yes. Because it’s wrong for her to be and he’s dressed just as this that he’s dressed and the same way—(Exp.: OK, good) as her.” (BPC: wrong for her to be X (*there*? *so critical*?)) [H.M. failed to conjoin a complement to the copular verb *to be*] H.M.: “and the fresh are not—are not. Doesn’t say that, it says not.” (BPC: the fresh fruit are not *for sale*) [H.M. failed to conjoin a complement to the copular verb *to be*] H.M.: “Since they’ve got their coffee already he isn’t—they just want their uh pie and the piece of this pie up here because the cake is down here.” (BPC: isn’t *in need of coffee*) [H.M. failed to conjoin a copular complement such as *in need of coffee* to *isn’t* in a coherent sentence plan with the surface elements *He isn’t in need of coffee*]
**Major Violations of Miscellaneous Conjunction Constraints**	
	H.M.: “If they don’t use legs like he does…” (BPC: use *their* legs) [H.M. failed to conjoin the possessive pronoun *their* to the noun *legs*] H.M.: “Because it’s too hard to do it that way.” (incomplete sentence) [H.M. failed to conjoin a main proposition to his subordinate *because* clause] H.M.: “And he has to use his legs to climb.” (incomplete sentence) H.M.: “And that man is trying to tell that woman not to sit there because it’s wet paint.” (incomplete sentence) H.M.: “I like some her … what she had.” (BPC based on the picture: I *would* like some *of* what she had) [H.M. failed to conjoin an auxiliary to *like* and omitted *of* in the complement *of what she had*]

BPCs are in parentheses. Square brackets enclose an explanation for typical examples in each category.

##### 4.2.1.3. Major Commission-Type CC Violations

Table 5 provides a complete list of major commission-type CC violations for H.M. and the controls, classified by syntactic category of the units involved. H.M. produced 1.35 commission-type CC violations per response *versus* a mean of 0.01 for the controls (*SD* = 0.04), a reliable 33.50 *SD* deficit.

**Table 5:** Major commission-type violations of CCs in Study 2 organized by type of constraint.

**Major Violations of Pronoun-Antecedent Conjunction Constraints**	
	H.M.: “David wanted him to fall and to see what lady’s using to pull himself up besides his hands.” (BPC: pull *herself* up besides *her* hands) [H.M. failed to conjoin the pronouns *herself* and *her* as appropriate for the antecedent *lady*] H.M.: “If they don’t use legs like he does … and his hands, they could fall.” (BPC: and *their* hands) [H.M. failed to conjoin the possessive pronoun *their* to the noun *hands* as appropriate for the antecedent *they*] H.M.: “I want that job … and … but she says, he gotta do the other part first.” (BPC 1, as appropriate for the antecedent *I want*: *I* gotta do the other part; or BPC 2, as appropriate for *he gotta*: *He* wants that job) H.M.: “this is just little things … éclairs.” (BPC: *these* *are* just little things)
**Major Violations of Pronoun-Referent Conjunction Constraints**	
	H.M.: “David wanted him to fall and to see what lady’s using to pull himself up besides his hands.” (BPC 1: what *she*’s using to pull *herself* up; or BPC 2, because the picture shows only men: what *this man’s* using) [H.M. failed to conjoin the appropriate reflexive pronoun to the antecedent *lady*; or H.M. failed to conjoin the appropriate common noun to the male referent doing the climbing] H.M.: “Is it crowded and it just pointed out this bus is up here.” (BPC based on the picture: *she* just pointed out) [H.M. failed to conjoin the pronoun *she* to the pictured female referent who is pointing] H.M.: “Since they’ve got their coffee already he isn’t—they just want their uh pie and the piece of this pie up here because the cake is down here.” (BPC for inaccurate pronoun use 1, *they* don’t already have *their* coffee is inaccurate because the picture shows only a woman with coffee: *she’s got her coffee*; BPC for inaccurate pronoun use 2, *they* don’t want *their* pie because the woman already has pie on her tray in the picture: *he just wants his pie*) H.M.: “Well he’s putting the price of it and price of thing what it is and she wants to in there and he’s waitin’ to be waited on.” (BPC based on the picture: *she*’s waitin’ to be waited on) H.M.: “Melanie gets on that one if she can and she wants her to travel along with him.” (BPC 1 based on the picture: *she* wants *him* to travel along with *her*; or BPC 2 based on the picture: *he* wants *her* to travel along with *him*)
**Major Violations of Subject-Verb Conjunction Constraints**	
	H.M.: “I don’t want to do it the same way as he do because you can’t do it that way.” (BPC 1: as he *did*; or BPC 2: as he *does*) [H.M. failed to conjoin the past tense marker to the verb *do*; or H.M. failed to conjoin the appropriate present tense singular marker to the verb *do*] H.M.: “Melanie tra … on that bus, the scrawny bus and have it drive it off … it, it drives it off.” (BPC 1 based on the picture: Melanie … *has* it drive … off; or BPC 2 based on the picture: Melanie … *has* him drive it off) [H.M. failed to conjoin the appropriate pronoun as subject in a sentence plan containing the verb *drive*] H.M.: “A driving wanna drive some place and this bus is stopped up there.” [BPC based on the picture: *they* wanna] Control participant: “one boy say” (BPC: *one boy says*)
**Major Violations of Miscellaneous Conjunction Constraints**	
	H.M.: “David wanted him to fall and to see what lady’s using to pull himself up besides his hands.” (BPC, because the picture shows only men: what *this man’s* using) [H.M. failed to conjoin the appropriate common noun to the male referent doing the climbing] H.M.: “Because it’s wrong for her to be and he’s dressed just as this that he’s dressed” (Non-causal use of *because*) H.M.: “And that man is trying to tell that woman not to sit there because it’s wet paint.” (BPC 1: *because the paint is wet*;or BPC 2: *because that’s wet paint*) H.M.: “I like some her…what she had.”(BPC for *her*: *of what she had*) [H.M. substituted *her* for *she*] H.M.: “Well this pie is- or the pie here was back here- and uh coffee is in there because heat a solid” (non-causal use of *because*) H.M.: “She doesn’t got any shoes on either.” (BPC 1: doesn’t *have* any shoes on; or BPC 2: hasn’t *got* any shoes on) [H.M. failed to conjoin the appropriate verb *have* to the auxiliary verb *do*; or H.M. failed to conjoin the appropriate auxiliary verb *have* to the verb *got*] H.M.: “Melanie tra … on that bus, the scrawny bus and have it drive it off…it, it drives it off.” (BPC for *scrawny*, which inaccurately describes two identical buses in the picture, one of which is *further away* or *more distant* but not *smaller* than the other: the *more distant* bus) [H.M. failed to conjoin the complex adjective *more distant* to the NP *more distant* *bus*] H.M.: “Melanie tra … on that bus, the scrawny bus and have it drive it off … it, it drives it off.” (BPC 1: Melanie … *has* it drive off; or BPC 2: Melanie … *has* him drive it off) H.M.: “I like some her … what she had.” (BPC based on the picture: *I would like some of what she had*) [H.M. substituted *her* for *she* in the complement *of what she had*] H.M.: “He hadn’t got any milk there or put it in his cup.” (BPC 1: he *hasn’t* got any; or BPC 2: hadn’t *gotten* any) H.M.: “I want that job … and … but *she* says, *he* gotta do the other part first.” (BPC: he*’s* gotta do) Control participant: “it has my size” (BPC: it *is* my size)
**Major Violations of Correlative Conjunction Constraints**	
	H.M.: “I … she wants the house painted the same as him and he wants to mow the lawn.” (BPC 1: as he *does*; or BPC 2: as *his house*) H.M.: “Yes. Because it’s wrong for her to be and he’s dressed just as this that he’s dressed and the same way—(Exp.: OK, good) as her.” (BPC: he’s dressed just as this *man is dressed*) H.M.: “Once has to be trash in yellow (inaudible) … is not here. (H.M. misread the target word *nor* as *not*) (Exp.: It says *nor*) She doesn’t want her pie.” [H.M. failed to use *nor* as requested] H.M.: “I want some of that pie either some pie and I’ll have some.” (misuse of *either*) H.M.: “Any pie to either have.” (misuse of *either*) H.M.: “Any pie that either she either had.” (two misuses of *either*)

BPCs are in parentheses, with numbers labeling alternative BPCs and multiple errors. Square brackets enclose an explanation for typical examples in each category.

#### 4.2.2. Specific Analyses: Gender, Number, and Person CCs

As applied to the TLC, gender, number, and person CCs refer to the fact that (a) proper names, pronouns, common nouns, and common noun NPs must agree in gender, number, and person with their referents in a picture, and (b) pronouns must agree in gender, number, and person with their antecedents in a sentence. 

##### 4.2.2.1. Gender, Number, and Person CCs for Proper Name Referents

H.M. produced seven proper names *versus* a mean of 0.0 for the controls, a reliable 6.0 *SD* difference by convention. For these seven proper name uses, H.M. violated 0 referent-proper name CCs involving gender, *versus* a mean of 0.0 for the controls (with *N* = 0 and *SD* = 0). For example, in the corresponding TLC pictures, H.M.’s proper names *Gary* and *David* in (23ab) referred to males, and *Melanie* in (23c) referred to a female.

(23a).
  H.M.: “Gary is … almos … almost … hasn’t been cut the same way.”
  (*Gary* is aninvented proper name that specifies an unknown man in the TLC picture)
(23b).
  H.M.: “*David* wanted him to fall and to see what lady’s using to pull himself up besides his hands.”
  (*David* is an invented proper name that specifies an unknown man in the TLC picture)
(23c).
  H.M.: “Melanie gets on that one if she can and she wants her to travel along with him.”
  (*Melanie* is an invented proper name that specifies an unknown woman in the TLC picture)

Analyses of referent-proper name CCs for person and number replicated and extended these gender CC results: H.M. produced 0 violations of referent-proper name CCs for person (with *N* = 7), *versus* a mean of 0.0 for the controls (with *N* = 0 and *SD* = 0), and 0 violations of referent-proper name CCs for number (with *N* = 7), *versus* a mean of 0.0 for the controls (with *N* = 0 and *SD* = 0). 

##### 4.2.2.2. Gender, Number, and Person CCs for Pronouns and Common Nouns

H.M. violated 22 person, number, and gender CCs involving pronouns and common nouns, *versus* a mean of 0.0 for the controls (*SD* = 0), a reliable 6.0 *SD* difference by convention. Of these, 14 were violations of gender CCs, as in (24).

(24).
  H.M.: “…to see what lady’s using to pull himself up besides his hands.”
  (BPC: *to see what this lady’s using to pull herself up besides her hands*; see Table 5 for H.M.’s complete utterance)
(25).
  H.M.: “If they don’t use legs like he does…and his hands…”
  (BPC: *If they don’t use their legs like he does … and their hands*; see Table 5 for H.M.’s complete utterance)
(26).
  H.M.: “it just pointed out this bus is up here.”
  (BPC based on the picture: *she just pointed out this bus is up here*; see Table 5 for H.M.’s complete utterance)
(27).
  H.M.: “she wants her to travel along with him.”
  (BPC: *she wants **him** to travel along with **her*** or *he* *wants* ***her*** *to travel along with* **him**; see Table 5 for H.M.’s complete utterance)

Example (24) contains two uncorrected CC violations involving the gender (male *versus* female) for pronoun antecedents: To agree in gender with their antecedent *lady*, H.M.’s pronouns *himself* and *his* in (24) should read *herself* and *her*. 

H.M.’s immediately subsequent utterance in (25) illustrates two additional uncorrected CC violations involving pronoun-antecedent number (singular *versus* plural): To agree in number with their antecedent *they*, H.M.’s pronouns in (25) should read ***they****… use **their** legs … and **their** hands …*

When using pronouns to designate people in TLC pictures (see Table 5), H.M. also violated 8 CCs involving the gender, number, and person for the referents of pronouns, *versus* a mean of 0.0 for the controls (*SD* = 0), a reliable 6.0 *SD* difference by convention. For example, H.M.’s “it just pointed out” in (26) violates a pronoun-referent CC for person because the pronoun *it* is inappropriate for referring to people. H.M. then produced two similar CC violations involving pronoun-referent gender in (27), his immediately subsequent utterance: Because a man and a woman (conversing in the picture) are the only possible referents for H.M.’s “she”, (27) should read either *she wants **him** to travel along with **her*** or ***he** wants **her** to travel along with him*. 

##### 4.2.2.3. CCs Involving Common Noun NPs

(28).
  H.M.: “it’s crowded school bus.”
  (BPC: *it’s a crowded school bus*; violation of a determiner-common noun CC; see Table 4 for H.M.’s complete utterance)
(29).
  H.M.: “and the fresh are not- are not…”
  (BPC based on TLC picture: *the fresh fruit are not*…; major violation of a modifier-common noun CC; see Table 4 for H.M.’s complete utterance)

Analyses of CC violations involving common noun NPs were relevant to the possibility that H.M. used proper names (e.g., *Gary*) to compensate for difficulties in forming functionally equivalent NPs (e.g., *this man*), even though proper name usage allowed no similar CC violations. H.M. produced 9 major omission-type CC violations involving determiner- and modifier-common noun NPs, *versus* a mean of 0.25 for the controls (*SD* = 0.53), a reliable 16.5 *SD* difference (see Table 4, Table 5). For example, (28) illustrates an omission-type CC violation in a determiner-common noun NP: H.M.’s uncorrected “it’s crowded school bus” (for BPC *a crowded school bus*) reflects omission of the determiner *a.* Similarly, (29) illustrates an omission-type CC violation involving a modifier-common noun NP: H.M.’s uncorrected “the fresh are not” (for BPC *the fresh fruit are not*) is ungrammatical because adjectives such as *fresh* require a noun such as *fruit* to complete the NP. 

### 4.3. Subsidiary Results

#### 4.3.1. H.M.’s TLC Proper Names: Retrieved or Invented?

Why did H.M. choose one proper name rather than another to refer to the unknown people in TLC pictures? One possibility is that prior to his lesion H.M. had already formed the appropriate referent-proper name links for referring to these TLC people because they reminded him of pre-lesion acquaintances. Under this hypothesis H.M. could therefore retrieve these referent-proper name links from memory rather than forming them anew. 

To test this hypothesis, we searched the 182-page Marslen-Wilson [5] transcript for the names that H.M. used on the TLC, e.g., *Melanie*, *David*, *Gary*, *Mary*, and *Jay*. We reasoned that if H.M.’s TLC names referred to pre-lesion acquaintances, he was likely to use their names when discussing pre-lesion acquaintances in Marslen-Wilson. However, our search results did not support this hypothesis: Although H.M. used many first names in Marslen-Wilson, e.g., *Arlene*, *George*, *Calvin*, *Tom*, *Robert*, *Franklin*, and *Gustav*, none matched his TLC names. This finding suggests that H.M. invented his TLC names and formed their referent-gender links anew rather than retrieving them on the basis of resemblance to past acquaintances. 

#### 4.3.2. Trouble Accompanying H.M.’s Use of Proper Names

A subtle type of trouble accompanied H.M.’s use of proper names in Study 2: Speakers using proper names to refer to someone unknown to their listeners normally add an introductory preface such as *Let’s call this man David*, and the many available collections of speech errors and malapropisms record no failures to produce such prefaces in memory-normal speakers (see, e.g., [50,51,52]). However, this unusual type of proper name malapropism was the rule for H.M.: none of his TLC proper names received introductory prefaces (see e.g., (23a–c)). 

Why did H.M. choose this flawed proper name strategy over the “deictic” or pointing strategy that memory-normal controls adopted in Study 2? Using this pointing strategy, controls described a TLC referent with a pronoun (e.g., *he*) or common noun NP (e.g., *this man*) while pointing at the picture so as to clarify their intended referent (necessary because TLC pictures always contained several possible human referents). 

Perhaps H.M.’s flawed proper name strategy reflects insensitivity to referential ambiguities, consistent with his well-established problems in comprehending the two meanings of lexically ambiguous sentences, e.g., performing at chance levels and reliably worse than controls in MacKay, Stewart *et al.* ([13]; see also [12] for a replication). This insensitivity would explain why H.M. used *David* without correction in (23b), even though *David* could refer to any of three unknown males in the TLC picture (a referential ambiguity that pointing would have resolved). 

Another (not necessarily mutually exclusive) possibility is that H.M. tried and rejected a deictic (pointing) strategy in (23b) because of the problems it caused. Under this hypothesis, H.M. was trying to say “David wanted this man to fall and to see what *he*’s using to pull *himself* up besides *his* hands” in (23b), but instead said “David wanted him to fall and to see what lady’s using to pull himself up besides his hands”, substituting the inaccurate and referentially indeterminate *lady* for the common noun *man*, omitting the demonstrative pronoun *this* in the deictic expression *this* *lady*, and rendering his subsequent pronouns, *himself* and *his*, gender-inappropriate for the antecedent *lady*. In short, by attempting to use the deictic strategy in (23b), H.M. ran into four types of trouble that he apparently tried to minimize by opting for a subtler (minor rather than major) “error”: use of proper names to describe unknown and un-introduced referents. 

### 4.4. Discussion

To summarize the main results of Study 2A, H.M. produced reliably more proper names than the controls on the TLC, and violated no CCs for gender, person, or number for any of his proper names. However, per TLC response, H.M. violated reliably more gender, person, and number CCs than the controls for the common noun antecedents of pronouns and for the referents of pronouns and common nouns, and he omitted reliably more common nouns, determiners, and modifiers than the controls when forming common noun NPs.

These results indicate that H.M. can conjoin referents with proper names of the appropriate person, number, and gender without difficulty, but he produces encoding errors when conjoining referents and common noun antecedents with pronouns of the appropriate person, number, and gender, and when conjoining referents with common nouns of the appropriate person and gender. 

This contrast between H.M.’s encoding of proper names *versus* pronouns and common nouns comports with the working hypothesis outlined earlier: Under this hypothesis, H.M. overused proper names relative to memory-normal controls when referring to people in MacKay *et al.* [2] because (a) his mechanisms are intact for conjoining the gender, number, and person of an unfamiliar person (or their picture) with proper names, unlike his corresponding mechanisms for pronouns, common nouns, and NPs with common noun heads, and (b) H.M. used his impaired encoding mechanisms for proper names to compensate for his impaired encoding mechanisms for the only other ways of referring to people: pronouns, common nouns, and common noun NPs. 

H.M. also omitted reliably more determiners when forming NPs with common noun heads, but these difficulties were not limited to determiners: H.M. also omitted reliably more modifiers and nouns in NPs with common noun heads. Present results therefore point to a general difficulty in encoding NPs, consistent with the hypothesis that H.M. overused his spared encoding mechanisms for proper names to compensate for his impaired encoding mechanisms for forming common noun NPs. 

## 5. Study 2B: How General are H.M.’s CC Violations?

To summarize, in Study 1, H.M. produced reliably more word- and phrase-level free associations than the controls, ostensibly in order to compensate for his difficulties in forming phrases that are coherent, novel, accurate, and grammatical. Then relative to controls referring to people in Study 2A, H.M. violated reliably more gender, number, and person CCs when using pronouns, common nouns, and common noun NPs, but not when using proper names. 

Following up on these results, Study 2B tested the Study 1 assumption that forming novel phrases that are coherent, accurate, and grammatical is *in general* difficult for H.M. This being the case, we expected reliably more encoding errors for H.M. than memory-normal controls in Study 2B across a wide range of CCs not examined in Study 2A, e.g., verb-modifier CCs (e.g., copular verbs cannot take adverb modifiers, as in *Be happily*), verb-complement CCs (e.g., verb complements such as *for* *her to come home* are required to complete VPs such as *asked for her to come home*), auxiliary-main verb CCs (e.g., the past participle *got* cannot conjoin with the auxiliary verb *do* as in *He doesn’t got it*), verb-object CCs (e.g., intransitive verbs cannot take direct objects, as in *The earthquake happened the boy*), modifierCCs (e.g., in non-metaphoric uses, adjectives cannot modify an inappropriate noun class, as in *He has thorough hair*), subject-verb CCs (e.g., in American uses, subjects and verbs cannot disagree in number, as in *Walmart sell it*), propositional CCs (e.g., *because* cannot conjoin causally unrelated propositions, as in *Because he has a name*, *they named him*), and correlative CCs (e.g., a member of one correlative conjunction pair cannot conjoin with a member of another pair, as in *She either likes him nor hates him*). 

### 5.1. Results

Excluding CC violations involving the gender, number, or person of pronouns, common nouns, and common noun NPs referring to people, H.M. violated 29 additional CCs, *versus* a mean of 0.25 for the controls (*SD* = 0.25), a reliable 114 *SD* difference. Subsequent sections report separate analyses of CC violations for verb-modifier CCs, verb-complement CCs, auxiliary-main verb CCs, verb-object CCs, modifier-noun CCs, subject-verb CCs, and correlative CCs. 

#### 5.1.1. CC Violations Involving Verb Complements or Modifiers

Overall H.M. violated 3 copular complement CCs (see Table 4), *versus* a mean of 0.0 for the controls (*SD* = 0). Example (30) illustrates one such CC violation involving the verb *to be*: H.M.’s “for her to be” in (30) is ungrammatical, reflecting uncorrected omission of a copular complement for the verb *to be*.

(30).
  H.M.: “Because it’s wrong for her to be…”
  (BPC based on the picture and utterance context: *it’s wrong for her to be there*: omission of a verb complement or modifier; see Table 4 for H.M.’s complete utterance)

H.M.’s difficulties in conjoining complements with the verb *to be* were not unique to the TLC. Note that H.M. produced remarkably similar uncorrected copular complement omissions on the TLC in (30) and during conversational speech in (31), in both cases yielding overall utterances that were incoherent, ungrammatical, and difficult-to-comprehend.

(31).
  H.M. (spontaneous conversation in [53]): “What’s found out about me will help others be.”
  (copular-complement CC violation)

#### 5.1.2. Violations of Auxiliary-Main Verb CCs

Example (32) illustrates a violation of an auxiliary-main verb CC, with two candidates tied for BPC: *she **doesn’t have** any shoes on* (where the verb *got* in H.M.’s “doesn’t got” is in error), and *she **hasn’t got** any shoes on* (where the auxiliary *do* in “doesn’t got” is in error) [54].

(32).
  H.M.: “She doesn’t got any shoes on…”
  (BPC: *she **doesn’t have** any shoes on* or *she **hasn’t got** any shoes on*; see Table 5 for H.M.’s full utterance)

#### 5.1.3. Violations of Verb-Object CCs

Example (33) illustrates a violation of a verb-object CC: H.M.’s “he’s trying to sell” is ungrammatical because transitive verbs such as *sell* require an object such as *it* (see Table 4 for other violations of verb-object CCs).

(33).
  H.M.: “…she’s taking that suit and he wants to take it … and he’s trying to sell.”
  (BPC based on the picture and utterance context: *trying to sell it*; major violation of a verb-object CC; see Table 4 for H.M.’s complete utterance)

#### 5.1.4. Violations of Modifier-Noun CCs

Example (34) illustrates a violation of a modifier-common noun CC because the adjective *scrawny* cannot modify inanimate nouns such as *bus* except in metaphoric uses such as personification [55]. However, metaphoric use of *scrawny* is implausible here because H.M. exhibits special problems with metaphors, performing at chance levels and reliably worse than controls in comprehending metaphors on the TLC (see [12]). Moreover, consistent with *scrawny* as a CC violation, H.M.’s *scrawny* is erroneous in other ways: The picture for (34) shows two identical buses, one of which is farther away or more distant but not smaller than the other (see Table 5).

(34).
  H.M.: “Melanie tra … on that bus, the scrawny bus.”
  (BPC based on the picture: *on that more distant bus*; see Table 5 for H.M.’s full utterance)

#### 5.1.5. Violations of Subject-Verb CCs

Because subjects and verbs must agree in number and person in grammatical English sentences, H.M.’s uncorrected “as he do” in (35) violates a number agreement CC (BPC: *as he does*). In (36), H.M.’s uncorrected “have it drive it off” violates a person CC and should read either *have **him** drive it off* or *have **her** drive it off* because the verb *drive* requires a human subject (personification aside, as discussed earlier; see Table 5 for H.M.’s complete utterance). Overall, H.M. violated 3 subject-verb CCs for number and person *versus* a mean of 0.13 for the controls (see Table 5).

(35).
  H.M.: “I don’t want to do it the same way as he do.”
  (BPC: *as he does*; see Table 5 for H.M.’s complete utterance)
(36).
  H.M.: “have it drive it off.”
  (BPC based on the picture: *have **him** drive it off* or *have **her** drive it off*; see Table 5 for H.M.’s complete utterance)

#### 5.1.6. Violations of Correlative CCs

Correlative conjunction occurs in grammatical sentences when speakers conjoin two equivalent syntactic structures (e.g., two nouns, two verbs, two NPs, two VPs, two prepositional phrases, or two propositions) via correlative conjunction pairs, e.g., *either-or*, or *both-and*, as in examples (37a–e).

(37a). ***Both*** *men **and ** women* *came* (nominal correlative conjunction)
(37b). *They****both*** *noticed **and** objected* (verbal correlative conjunction)
(37c). ***Either*** *the man **or** his wife* *came* (NP correlative conjunction)
(37d). *He****neither*** *noticed the error **nor** corrected* *it* (VP correlative conjunction)
(37e). *They met****either*** *in the garden **or** in the house* (PP correlative conjunction)
(37f). ***Either*** *Mary came **or** she went home* (propositional correlative conjunction)

We scored major violations of correlative CCs when speakers used one or both members of a correlative conjunction pair in uncorrected utterances that were inaccurate, ungrammatical, or both, as in examples (38)–(40). The *either-or* BPC in (38) conjoins the propositions *any pie that she had* and *any pie that she wanted*, but H.M. repeated *either* and omitted *or* and its associated proposition without correction. The *either-or* BPC in (39) conjoins the VPs *want some of that pie* and *will have some cake*, but H.M. omitted *or* and *cake* in *have some cake*.The *either-or* BPCin (40) conjoins the verbs *have* and *eat*, but H.M. omitted *or* and *have* (see Table 5). Overall H.M. violated five correlative CCs, *versus* a mean of 0.0 for the controls (*SD* = 0), a reliable 6.0 *SD* difference by convention.

(38).
  H.M.: “Any pie that either she either had.”
  (BPC: *He didn’t want any pie that she either had or wanted*)
(39).
  H.M.: “I want some of that pie either some pie and I’ll have some.
  (BPC: *I* *either want some of that pie or I’ll have some cake*)
(40).
  H.M.: Any pie to either have. (BPC: *He didn’t want any pie to either have or eat*)

H.M. also had problems defining, comprehending and reading the correlative conjunctions *either-or* and *neither-nor*. In (41a), H.M. inaccurately defined *either* as “or” (although associated with *or* in semantic memory, *either* links alternative possibilities but does not mean *or*). It was as if H.M. responded “or” via phrase-level free association without comprehending *either* as an isolated word. Similarly in (41b), H.M. failed to distinguish *or* *versus* *nor* as concepts, defining *nor* as “Or she could say this.”

(41a).
  H.M. (in response to an experimenter question about what the word *either* means): “Or.”
  (BPC: *Either refers to alternative possibilities*)
(41b).
  H.M. (in response to the experimenter’s request to define the correlative conjunction *nor*): “Or she could say this.”
  (BPC: *Nor refers to negation or non-occurrence of an additional event or possibility*)

Turning to correlative conjunction reading errors, H.M. misread the target word *nor* as *not* once in (42) (without correction, despite the experimenter’s “It says *nor*”), and twice without correction in (43) (despite admitting “Doesn’t say that”, H.M. again misread *nor* as *not*). Both uncorrected reading errors suggest inability to distinguish the concepts *nor versus not*.

(42).
  H.M.: “Once has to be trash in yellow (inaudible) … is not here. (Here H.M. substituted *not* for the target word *nor*) (Exp.: “It says *nor*.”) She doesn’t want her pie.”
  (H.M. failed to use *nor* as per the TLC instructions and experimenter reminder)
(43).
  H.M.: “Well you—she wants one thing and he wants another thing and the fresh are *not*—are *not*. Doesn’t say that, it says *not*.”
  (BPC: *Doesn’t say that*, *it says nor*; see the supplementary materials for H.M.’s complete utterance)

### 5.2. Discussion

Besides the six types of CC violations examined in Study 2A, H.M. violated more than seven additional types of CCs reliably more often than the controls during sentence planning in Study 2B (see also the major violations of miscellaneous CCs in Table 4, Table 5). Overall, H.M. violated common noun-antecedent CCs, common noun-referent CCs, pronoun-antecedent CCs, pronoun-referent CCs, determiner-common noun CCs, modifier-common noun CCs, verb-modifier CCs, auxiliary-main verb CCs, verb-object CCs, modifier-noun CCs, subject-verb CCs, propositional CCs, and correlative CCs. These CC violations indicate extensive damage to category-specific encoding mechanisms for rapidly linking a wide range of linguistic and non-linguistic (referential) units for creating accurate, coherent, and grammatical phrases. 

H.M.’s violations of correlative conjunction CCs (involving, e.g., *either*/*or* and *both*/*and*) are especially relevant to his non-use of correlative conjunctions in MacKay *et al.* [2]. H.M. didn’t fail to use correlative conjunctions because he couldn’t retrieve this category of words: When violating correlative conjunction CCs, H.M. produced the first but not the second member of correlative conjunction pairs, indicating a problem in encoding the proposition, NP, or VP that must follow his initial correlative conjunctions. 

#### 5.2.1. Theoretical Significance of H.M.’s CC Violations

Present results indicate a link between hippocampal region damage and two types of encoding errors: omission-type and commission-type. Omission-type encoding errors violate CCs because a concept or unit that should become conjoined in an internal representation is omitted, and the item-to-item sequential associations postulated in several theories (beginning with [56]) represent one possible hippocampal region binding process that breaks down to yield omission-type CC violations. Under item-to-item sequential theories, H.M. produced omission-type encoding errors such as “the fresh are not…” rather than “the fresh *fruit* are not...” because his damaged hippocampal region failed to bind *the* as an item to the next item in the intended sequence, *fruit*. However, item-to-item sequential associations cannot account for reverse-sequence CC violations, where a prior item is omitted, as when *would* is omitted in the intended sequence *would like*, or *this* is omitted in the intended sequence *this lady* (see Table 4 for additional reverse-sequence omission-type CC violations).

Commission-type encoding errors violate CCs by conjoining concepts or units that should not be conjoined, and challenge the larger category of theories that postulate item association (without regard to sequence). Under these theories, encoding errors such as “to see what she’s using to pull himself up” instead of *to see what she’s using to pull herself up* reflect failure, not to conjoin sequential strings such as *what she’s using* and *to pull herself up*, but to conjoin a specific item (here, *herself*). However, the regularities in H.M.’s many CC violations involving the pronoun category suggests that these errors reflect not failures to conjoin specific items (here, *herself*), but to conjoin underlying units representing abstract concepts such as *female* *third person singular* that determine the surface form, here, *herself* as the context-appropriate reflexive pronoun (rather than *himself* or *themselves*) for the subject *she*. Encoding errors such as “the same way as he do” instead of *the same way as he does* likewise reflect failure, not to conjoin *he* and *does* as lexical items, but to conjoin units representing abstract concepts, such as *third person singular* for determining *does*, as the context-appropriate verb form. Encoding errors such as “the fresh are…” instead of *the fresh fruit are …* likewise reflect failure to conjoin the familiar unit *fresh* in the abstract category ADJECTIVE with the familiar unit *fruit* in the abstract category NOUN to form *the fresh fruit*, a new unit in the abstract category NP. Finally, H.M.’s encoding errors for one set of abstract categories (COMMON NOUNS, PRONOUNS, and COMMON NOUN NPs), but not another (PROPER NAMES), indicate that when binding units into chunks (including but not restricted to higher level phrase and propositional units), hippocampal region encoding mechanisms for language and memory must operate on abstract concepts and categories (such as proper names), rather than items (individual words such as *David*).

H.M.’s CC violations therefore raise three important questions not adequately addressed in current binding theories: (a) By what regulatory or control mechanisms does the hippocampal region specify what *categories* of units can and cannot become conjoined? (b) What are the underlying units in these categories and how do they become conjoined? and (c) How does the hippocampal region ensure that one *category* of units successfully conjoins with another (rather than being omitted)? Complete and adequate accounts of the brain mechanisms underlying normal speech production therefore await theoretical answers to these questions. 

Moreover, complete and adequate accounts of episodic memory also await theoretical answers to analogous questions because amnesics with hippocampal region damage produce similar CC violations in immediate memory tasks. For example, patients with hippocampal damage falsely classify new or never previously experienced conjunctions of memory components as ‘‘old’’ or actually experienced reliably more often than memory-normal controls in verbal and visual episodic memory tasks (see [57]; also [58]). As Kroll *et al.* [57] point out (p. 176), directly deriving such illusions from “our current theories of the cognitive and neural basis of memory processes” is “the central problem for today”, and remains a central problem now, over 17 years later. 

#### 5.2.2. Memory Deficits for Episodic and Semantic Information: An Alternate Account?

According to Duff and Brown-Schmidt [59], the language deficits of amnesics are side effects of their episodic and semantic memory deficits. Because this hypothesis is relevant to H.M.’s CC violations and other language deficits, we therefore discuss the general plausibility of the Duff Brown-Schmidt hypothesis and its associated evidence. 

##### 5.2.2.1. Evidence Consistent with the Duff Brown-Schmidt Hypothesis

Duff and Brown-Schmidt [59] suggested that a separate (non-linguistic) episodic memory system underpins language use, especially the creative retrieval and binding of visual and linguistic information. Evidence for this hypothesis came from errors in the two-person communication game in Duff *et al.* [4], where amnesics and memory-normal controls were forced to repeatedly discuss the same objects: Unlike the controls, the amnesics often violated a CC by using *a* rather than *the* to describe previously discussed objects. Because the Duff *et al.* [4] amnesics by definition had episodic memory problems, Duff *et al.* therefore assumed that their episodic memory problems involving non-linguistic “information about the co-occurrences of people, places, and objects along with the spatial, temporal, and interactional relations among them” caused their *a-*for*-the* substitutions (p. 672).

However, the Duff Brown-Schmidt hypothesis does not adequately explain H.M.’s determiner errors because: (a) mentioning previously discussed objects or episodes was unnecessary on the TLC (unlike in [4]); (b) H.M. produced no more encoding errors for *a*/*the* than for other determiners (e.g., *this*, *some*) that are a-historic and independent of episodic memory (see Table 4); and (c) all of H.M.’s *a*/*the* errors involved omission of *a* or *the* (see Table 4), rather than substitution of one for the other (as in [4]). 

Of course, H.M.’s problems with determiners other than *a*/*the* could reflect generalized avoidance of troubles caused by *a* and *the* under the Duff *et al.* [4] hypothesis. However, generalized avoidance predicts underuse of determiners relative to controls, an outcome not observed in MacKay *et al.* [2], and fails to predict the noun omissions that often *followed* H.M.’s (appropriately produced) determiners (see Table 4). 

##### 5.2.2.2. General Plausibility of the Duff Brown-Schmidt Hypothesis

Viewing non-linguistic episodic and semantic memory systems as central to the “creative use of language” and explaining language deficits in amnesia as due to deficits in non-linguistic declarative memory systems for retrieving and binding visual and linguistic information faces five challenges on the road to becoming a theory. First, extensive evidence indicates that H.M.’s basic problem lies not in retrieving pre-encoded information but in encoding or representing information anew (see Study 1; Study 2C; [2,24]). Second, vision-language bindings were not problematic for H.M. *in general*: Contrary to the Duff and Brown-Schmidt hypothesis, H.M. exhibited no difficulties when encoding vision-language bindings involving the gender, person, and number of the referents for proper names. Third, H.M.’s problems with language-language bindings (involving pronoun-antecedent, modifier-common noun, verb-modifier, auxiliary-main verb, verb-object, subject-verb, propositional, and correlative CCs): (a) closely resembled his vision-language binding problems (involving pronoun- and common noun-referents); (b) accounted for most of H.M.’s CC violations (see Table 4, Table 5); and (c) are not plausibly explained in terms of non-linguistic processes. Fourth, declarative memory explicitly involves *conscious* recollection of events and facts (see e.g., [60]), but no evidence, introspective or otherwise, indicates that conscious recollection underlies the creative everyday use of language. Indeed, extensive evidence indicates that creative language use can proceed unconsciously, and a simpler hypothesis with a great deal of support is that language use *per se* is creative, without help from non-linguistic memory systems (see e.g., [36,61]). Finally, no empirical results indicate that the sparing and impairment in H.M.’s non-linguistic (episodic memory and visual cognition) systems caused the sparing and impairment in his linguistic systems or vice versa. 

## 6. Study 2C: Minor Retrieval Errors, Aging, and Repetition-Linked Compensation

Study 2C had three goals. One was to re-examine the retrieval of familiar units (phrases, words, or speech sounds) on the TLC. Here our dependent variable (unlike in [2] and Study 1) was minor retrieval errors such as (6)–(8). Minor retrieval errors (a) include the sequencing errors that interested Lashley [1] and virtually every speech error researcher since then, and (b) occur when speakers substitute one phrase, word, or phonological unit (e.g., NP, noun, or vowel) for another unit in the same category (consistent with the sequential class regularity) without disrupting ongoing communication (because minor errors are corrected with or without prompting from a listener). We expected H.M. to produce reliably more minor retrieval errors than controls if his communication deficits reflect retrieval problems (contrary to assumptions in [2] and Study 1). However, we expected H.M. to produce no more minor retrieval errors than memory-normal controls if his communication deficits reflect encoding difficulties, as assumed in Study 2B.

As goal two, Study 2C examined four phenomena reliably associated with aging: dysfluencies, off-topic comments, neologisms, and false starts (see e.g., [62,63,64,65,66,67,68,69,70]). Under the hypothesis that H.M.’s communication deficits reflect exaggerated effects of aging, we expected H.M. to exhibit reliably more of these age markers than age-matched controls on the TLC. 

As goal three, Study 2C examined speech sounds, words, and phrases that participants repeated on the TLC. We expected reliably more word- and phrase-level repetitions for H.M. than the controls if repetition enables amnesics to form internal representations of novel information (see e.g., [6,7,8,9,10,11,12,13,14,15,16,17,18]), including novel phrase- and sentence-level plans. However, we expected no difference in speech sound repetition (stuttering) for H.M. *versus* memory-normal controls because repetition at phonological levels cannot compensate for H.M.’s inability to create novel phrase- and sentence-level plans.

### 6.1. Methods

Scoring and coding procedures resembled Study 2AB with two exceptions: First, to score minor retrieval errors, three judges (not blind to H.M.’s identity) received: (a) the TLC pictures and target words; (b) the transcribed responses of H.M. and the controls; (c) the definition of minor retrieval errors; and (d) typical examples unrelated to the TLC (e.g., (4), and (6)–(8)). The judges then used the definition and examples to mark minor retrieval errors on the transcribed responses, and when two or more judges marked the same error, it was recorded in a final transcript. 

Second, Study 2C analyzed the neologisms, false starts, dysfluencies, and off-topic comments that were eliminated from the transcripts in Studies 1–2 and MacKay *et al.* [2]. Neologisms included all non-standard pronunciations of a familiar word; dysfluencies were “um”s and “uh”s; off-topic comments were irrelevant remarks about the task or the experimenter (e.g., “How’s that suit you?”, where *that* refers to a self-produced response, and *you* to the experimenter); and false starts were sentence-level revisions or changes (excluding error corrections), where a speaker began with one plan or intended output, then shifted to another. For example, “they think it’s—they can’t do it because it’s too hard” was coded as a false start because the participant began to say *they think it’s too hard* but switched to “they can’t do it because it’s too hard”.

Finally, Study 2C determined the frequency of three types of repetition: stutters, unmodified word string repetitions, and elaborative repetitions. Following MacKay and MacDonald [71], stutters involved immediate repetitions of word-initial speech sounds, syllables, and words, e.g., “s—school” (repetition of a word-initial speech sound). Unmodified word string repetitions involved immediate repetition of a sequence of words without correction, as in “but it was, but it was”. Elaborative repetitions involved repetition of one or more *concepts* in distinctly different phrases. The repeated words italicized in (44) illustrate a stutter (*it*, *it*) and two elaborative repetitions (that *bus*, *the scrawny bus*, and *drive it off … it* *drives it off*”, where *drives* elaborates the concept *drive*). The repeated words italicized in (45) illustrate an unmodified word string repetition (it’s crowded … *it’s crowded*) and two elaborative repetitions (*it’s crowded* … too *crowded*, and to go on the bus … *to get on the bus*, *where get* elaboratesconceptual *go*). The repeated words italicized in (46) illustrate an elaborative repetition (this pie is … *the pie here was back here*, where *was* elaborates *is* as + past).

(44).
  H.M.: “Melanie tra … on that *bus*, *the scrawny bus* and have it *drive it off … it*, *it drives it off*.” (repeated words in italics)
(45).
  H.M.: …she wants to go on the bus … and it’s crowded … *it’s crowded* … Too *crowded* to get *on the bus*.
  (repeated words in italics)
(46).
  H.M.: “Well this pie is- or the *pie* here *was* (*is* + PAST) back *here*—”
  (brackets ours)

### 6.2. Results

H.M. produced no more minor word, morpheme, and phonological retrieval errors than the controls. The mean number of word and morpheme retrieval errors per response was 0.00 for H.M. and 0.00 for the controls (*SD* = 0.00), with absolute *N*s too small for meaningful statistical analysis. The only possible phonological retrieval error in the database was ambiguous: “Is it crowded” in (47) transposes either the phonological units /s/ and /t/ or the words *is* and *it* in the BPC *It is crowded*. However, this error was neither a minor phonological error nor a minor word retrieval error because (a) it was uncorrected, and (b) *it* and *is* belong to different lexical categories (pronoun and copular verb). The mean number of minor phonological sequencing errors was therefore 0.07 per response for H.M. *versus* 0.01 for the controls (*SD* = 0.04), a non-reliable 1.5 *SD* difference with *N*s too small for meaningful analysis.

(47).
  H.M.: “Is it crowded...”
  (BPC based on the picture: *It is crowded*)

#### 6.2.1. Age Markers: Neologisms, Dysfluencies, Off-Topic Comments, and False Starts

Age markers did not differ for H.M. *versus* the controls. The mean number of neologisms was 0.00 per TLC response for H.M. *versus* a mean of 0.03 for the controls (*SD* = 0.05), a non-reliable 0.60 *SD* difference with *N*s too small for meaningful analysis. Dysfluencies (“um”s and “uh”s) were no more common for H.M. than the controls. The mean number of “um”s per TLC response was 0.00 for H.M. *versus* 0.34 for the controls (*SD* = 0.52), a non-reliable difference. The mean number of “uh”s per TLC response was 0.10 for H.M. *versus* 0.48 for the controls (*SD* = 1.04), a non-reliable 0.37 *SD* difference. The mean number of off-topic comments per response was 0.10 for H.M. *versus* 0.36 for the controls (*SD* = 0.42), a non-reliable 0.63 *SD* difference. False starts or changes in an ongoing response (excluding error corrections) were no more common for H.M. than the controls. The mean number of false starts per response was 0.10 for H.M. *versus* 0.06 for the controls (*SD* = 0.07), a non-reliable 0.86 *SD* difference.

#### 6.2.2. Elaborative Repetitions, Stutters, and Unmodified Word String Repetitions

The mean number of elaborative repetitions per response was 0.25 for H.M. *versus* 0.04 for the controls (*SD* = 0.05), a reliable 4.20 *SD* difference. The mean number of stutters per response was 0.1 for H.M. *versus* 0.24 for the controls (*SD* = 0.21), a non-reliable 0.67 *SD* difference. The mean number of unmodified word string repetitions per response was 0.1 for H.M. *versus* 0.06 for the controls (*SD* = 0.07), a non-reliable 0.57 *SD* difference. 

### 6.3. Discussion

#### 6.3.1. Minor Retrieval Errors

H.M. produced no more minor retrieval errors involving phrases, words, or phonological units than the controls in Study 2C (see also [20,32]). These results suggest that H.M.’s mechanisms for retrieving and sequencing phrases in sentences, words in phrases, and phonological units in syllables are intact, consistent with (a) his undamaged frontal cortex (see [72]), and (b) extensive evidence indicating that retrieval mechanisms are localized in frontal areas, e.g., Chang *et al.* [73], where extremely localized high gamma (HG, 70–200 Hz) activity in the prefrontal cortex immediately preceded and apparently determined response-related retrieval of specific target phonemes (for additional evidence consistent with a frontal locus for retrieval mechanisms, see [74]).

#### 6.3.2. Age Markers: Neologisms, False Starts, Dysfluencies, and Off-Topic Comments

H.M. produced no more neologisms, false starts, dysfluencies and off-topic comments than memory-normal controls in Study 2C, results that rule out exaggerated effects of aging as the basis for H.M.’s communication deficits because these phenomena increase reliably with aging (see e.g., [62,63,64,65,66,67,68,69,70]). 

These findings, together with H.M.’s normal rate of minor retrieval errors, also rule out aphasia, because left hemisphere aphasics produce reliably more neologisms, dysfluencies, and retrieval errors than normal controls (see e.g., [75,76,77,78]). The close parallels between H.M.’s deficits in language and visual cognition (see [31]) also render implausible the hypothesis that H.M.’s language deficits reflect incipient or difficult-to-detect left- but not right-hemisphere white matter damage (see [72]). 

What then of the preliminary observations that raised the question of whether H.M. exhibits compound category-specific aphasia, with more neologisms, omissions, transpositions, perseverations, and anticipations of words, phrases, and phonological units than memory-normal controls (see MacKay *et al.* [2])? Close inspection indicates that spared retrieval mechanisms are consistent with these preliminary observations. First, H.M.’s omissions, transpositions, perseverations, and anticipations of words and phrases in MacKay *et al.* [24] were major (ungrammatical and uncorrected) *encoding* errors rather than minor *retrieval* errors that could in principle contradict intact retrieval mechanisms. Second, aphasics’ neologisms involve familiar words, e.g., *car* misproduced as “kike”, whereas H.M.’s neologisms involved low frequency (LF) words, e.g., *euphemism* misread as “embryism” (see [21]). Also unlike category-specific aphasics, H.M. produced no more neologisms overall and *fewer* neologism strings (e.g., “tralie”, “trassel”, “travis”, and “trussel” for *trellis*) than controls on the Boston Naming Test (see [32]). 

#### 6.3.3. Elaborative Repetitions, Stutters, and Unmodified Word String Repetitions

Relative to the controls, H.M. overproduced one type of repetition (elaborative repetitions) but not others (stuttering and unmodified word repetitions), and the question is why. The most plausible hypothesis is that H.M.’s elaborative repetitions reflect a deliberate strategy to offset his problems in forming new internal representations: By producing a familiar word or phrase and then intentionally repeating it with elaboration, H.M. was able to form internal representations for novel phrase- and proposition-level plans via repetition, one link at a time. 

Example (45) illustrates this elaborative repetition process: H.M. first produced the proposition “…it’s crowded” in (45) and then immediately repeated the verb *crowded* and added *too* as elaboration, which allowed formation of the VP “…too crowded” and avoided a major encoding error: *It’s crowded to get on the bus*. H.M.’s elaborative repetition strategy therefore had greater applicability than his proper name strategy, which applied to number, gender, and person marking in references to people (see Study 2A), but not to forming *any* new phrase- or proposition-level plan. 

As another contrast with elaborative repetitions, stuttering repetitions reflect involuntary re-activations of highly practiced phonological and muscle-movement units in preformed word- or phrase-level plans (see [79], pp. 157–197; [71]). As a consequence, H.M. produced no more stuttering repetitions than controls because his mechanisms for activating (retrieving) units that are pre-encoded and highly practiced are intact (as his normal rate of minor phonological retrieval errors suggests).

When did H.M. develop his elaborative repetition strategy? Close inspection of Marslen-Wilson [5] indicates that H.M.’s elaborative repetition strategy was well developed at age 44. For example, when responding to the question “Do you remember any of the kids there in kindergarten?” in (48a), H.M. produced five elaborative repetitions, unlike the typical control participant in (48b), who produced none when responding to the same question in MacKay *et al.* [22]. Like his proper name and free association strategies, H.M.’s elaborative repetition strategy therefore preceded middle age, was unrelated to age-linked cognitive decline, and may have originated in the 1950s as a way of offsetting effects of his hippocampal region damage.

(48a).
  H.M.: “Uh, just ... uh ... was a private kindergarten, and being on Burnside Avenue, and we ... my mother would take me down ... Burnside Avenue to ... uh ... can’t think of the name of the street … but where the drugstore was, and we’d gather there, *the kids*, *all of us kids* and then we’d ... the teacher that was ... taking us down to the private kindergarten, would take us down there ... that way and ... because there were ... from different areas and *that was one of the* ... *that was one of the main* ... *spots*, *the northern spot* ... I say northern but it isn’t northern, it’s east ... uh ... *the collect ... area*, *we’d gather at that area*, she’d take us down, then she’d have to go and ... her mother … would ... not she, her *mother* (emphasis in original) would be collecting the kids on … uh … the west ... so ... they’d come together and *meet ... naturally all meet in the same house*”.
  (elaborative repetitions in italics)
(48b).
  Typical control participant: “None in kindergarten. I don’t remember. I had, um ... cause I don’t know if it’s kindergarten, first grade. I remember a couple of other children.”
  (see text for explanation)

## 7. General Discussion, Conclusions, and Caveats

### 7.1. Impaired Planning Processes in Amnesia

Present results indicate that when referring to unfamiliar people, H.M. is unable to reliably encode (a) the gender, person, and number for pronouns, common nouns, and common noun NPs, and (b) a wide range of other constraints governing the conjunction of verbs and their modifiers, common nouns and their determiners, auxiliary verbs and their main verbs, verbs and their objects, subjects and their verbs, correlative structures, and subordinate propositions that modify a main clause. In short, H.M. experiences difficulty forming internal representations for most categories of novel information during sentence planning, consistent with the difficulties of other amnesics in planning for events that might occur in their personal future (see [80]). 

### 7.2. Spared Category-Specific Encoding Processes

Despite these difficulties, H.M. can create plans for producing at least one category of novel linguistic-referential information: the gender, person, and number of proper names for referring to unfamiliar people. As discussed next, this finding raises six interesting questions about encoding in language and other cognitive systems: (a) What other linguistic-referential encoding categories are spared in H.M.? (b) What are the general implications of selectively spared encoding processes? (c) Does H.M.’s visual cognition and episodic memory exhibit spared encoding categories? (d) Do other amnesics exhibit spared encoding categories? (e) How many category-specific mechanisms are needed to encode episodic and linguistic information? and (f) Why does H.M. detect and correct proper name errors but not other types of errors?

#### 7.2.1. Are Other Linguistic-Referential Encoding Categories Spared in H.M.?

Like proper names, numbers may be a spared linguistic-referential encoding category in H.M. First, H.M. retrieved specific numbers with remarkable frequency when discussing early childhood memories in Marslen-Wilson [5], e.g., the number 509 eleven times, the number 449 eight times, the number 63 four times, and the number 15 twice. Second, H.M. successfully recalled numbers in Marslen-Wilson that he could only have encountered many years after his lesion. For example, in (49), H.M. recalled that the English rock band Rolling Stones had five members in 1970.

(49).
  H.M. (describing a photograph of *the Beatles*):They’re not the Rolling Stones … no, I didn’t think they were because I thought there was five ... Rolling Stones.”

Further research therefore seems warranted to determine whether H.M. encodes and retrieves numbers but not common nouns relatively more often than memory-normal controls: For example, with numbers but not common nouns as a spared encoding category, H.M. should recall proportionally more unrelated numbers than nouns relative to age-matched controls in the immediate recall tasks of Drachman and Arbit [81].

#### 7.2.2. Why Are Some Encoding Categories Selectively Spared?

##### 7.2.2.1. The Ease-of-Encoding Hypothesis

The hypothesis that H.M. can successfully encode and recall proper names because this category is inherently easy to encode and retrieve can be rejected because extensive evidence indicates that proper names are *more* (rather than *less*) difficult to encode and retrieve than other types of information about people such as their (common noun) occupation (see e.g., [82,83,84]). 

##### 7.2.2.2. The Lesion-Specificity Hypothesis

Under the lesion-specificity hypothesis, H.M.’s hippocampal region damage (a) spared his category-specific mechanisms for encoding proper names, but (b) impaired functionally equivalent mechanisms for referring to people via NPs (that conjoin common nouns with determiners and other modifiers), and pronouns (that conjoin with antecedents in a sentence or with referents in a picture). 

#### 7.2.3. Does H.M.’s Episodic Memory Exhibit Similar Sparing?

Like his ability to encode proper name gender, number and person, H.M.’s ability to encode the (novel) time and place of particular topics of conversation may be spared, a point illustrated in (50), the fourth segment in the Marslen-Wilson [5] excerpt discussed in Section 1.1, segments (1–3).

(50).
  M-W.: Uh-huh ... right … um … How are you feeling? … tired?
  H.M.: Well … I’m just wondering myself … now ... well … when you fellows are taking this all down of course on tape … but I’m wondering just how it will be…
  M-W.: How do you mean, “how it will be”?
  H.M.: Well … just how I have spoken, how I sound, and what my answers are … and ... uh …a a big question mark right there…
  M-W.: Your answers are very helpful, very helpful indeed.
  H.M.: They are … I hope so…
  M-W.: But are you beginning to feel tired perhaps…?
  H.M.: Um?
  M-W.: Are you … are you at all tired? We’ve asked you a lot of questions.
  H.M.: No, I’m not tired.
  M-W.: Do you know what the time is?
  H.M.: Well, by this it says … twenty minutes of four (pointing at a clock).
  M-W.: Ah … well, what have you…what have we been asking you questions about?
  H.M.: Well about … the war, Chiang Kai Shek … the war ... China…
  M-W.: Before that?
  H.M.: And Indochina … and … about us... uh ... helping out ...

Note that H.M. recalled five prior topics in (50), one of which (“Indochina”) occurred over 100 s earlier (see (1.1)). Note also that this topic (a) concerned post-lesion events (the Vietnam war), (b) was not previously mentioned in his conversations with Marslen-Wilson [5], and (c) could not have been rehearsed during the 26 conversational turns and over 200 words of unrelated intervening discussion in (1–3) and (50) about what time it was, the tape recorded session, Chiang Kai Shek, China, how H.M. was now feeling, what he meant by the expression “how it will be”, whether he was “at all tired”, and how he was helping science by participating in this and other studies. 

H.M.’s successful recall of this novel topic after such a long interference-filled interval is remarkable because (a) following shorter intervals, H.M. has failed to recall other categories of personally experienced events, such as where and when he has met someone, and (b) H.M. is generally assumed to be “marooned in the present” and unable to recall novel events of any type following interference-filled intervals longer than about 18 s. Equally remarkable, this instance was not unique: H.M. successfully recalled other topics of conversation after interference-filled intervals at several other points in Marslen-Wilson [5] (see [22]). Under the lesion-specificity hypothesis, such feats of recall reflect sparing of H.M.’s hippocampal region mechanisms for encoding topics of conversation as episodic events, despite damage to his mechanisms for encoding many other types of personally experienced events. 

#### 7.2.4. Does H.M.’s Visual Cognition Exhibit Similar Sparing?

Like his ability to encode topics of conversation and proper names, H.M.’s ability to encode the size and orientation of (novel) visual patterns may also be spared. In the MacKay and James [31] hidden figure task, H.M. made more shape errors (tracing forms in a concealing array that differed in shape from the target), but no more size errors (tracing forms in a concealing array that matched the target in shape but not size), and no more orientation errors (tracing forms in a concealing array that matched the target in shape but not orientation) than the controls (albeit with *N*s too small for meaningful analysis). 

One possible interpretation of this (tentative or marginal) result (if replicable in other amnesics) is that complex but not simple processes are impaired in H.M. (because size and orientation intuitively seem simpler to represent than form). However, as Koch and Tononi [85] point out, processes that intuitively seem simple often aren’t. In particular, representing orientation must be complex because current computer programs cannot detect major orientation errors introduced into photographs of natural scenes (see [85]), unlike humans (including H.M.) in the “What’s-wrong-here” task.

Another possible interpretation of this result is that many different encoding mechanisms normally conjoin units for creating novel internal representations for visual patterns that the partial nature of H.M.’s hippocampal region damage (see [72]) may have impaired his mechanisms for encoding visual form while sparing his mechanisms for encoding size and orientation. Under this interpretation, H.M. exhibits category-specific impairment in sentence production, episodic memory, and visual cognition, reflecting damage to his mechanisms for encoding many but not all categories of novel episodic, linguistic, and visual information. 

#### 7.2.5. Do Other Amnesics Exhibit Spared Encoding Categories?

Under the lesion-specificity hypothesis, spared encoding categories can be expected to vary across amnesics with partial damage to the hippocampal region depending on the precise locus of damage, and consistent with such variability, some amnesics exhibit selective sparing for specific types of novel semantic information (unlike H.M.). An example is “Mickey”, a patient with little or no ability to recall a wide range of novel semantic and episodic information (see [86], pp. 165–166). However, when asked to learn the answers to novel trivia questions such as “Where was the first baseball game played?” (*Hoboken*), and “Who holds the world record for shaking hands?” (*Theodore Roosevelt*), Mickey successfully recalled these name-fact pairings 20 minutes later, despite inability to recall when or where he learned them. Although further research is needed to delineate the precise nature of (a) Mickey’s underlying brain damage, and (b) his spared *versus* impaired encoding categories, Mickey can clearly encode at least one type of new semantic information (the links between events and proper names) but not others, just as H.M. can encode some types of new episodic and linguistic-referential information but not others. 

#### 7.2.6. H.M.’s Language and Memory: How Plausible Is the Lesion-Specificity Hypothesis?

Three factors discussed next add plausibility to the lesion-specificity account of H.M.’s selectively spared and impaired encoding categories: (a) the partial nature of his hippocampal region damage; (b) how many unique category-specific encoding mechanisms must reside in the hippocampal region; and (c) other sources of evidence for selectively spared and impaired encoding categories. 

##### 7.2.6.1. The Partial Nature of H.M.’s Hippocampal Region Damage

It makes sense that some of H.M.’s category-specific encoding mechanisms remained intact because (a) the hippocampal region is the most plausible locus for linguistic and non-linguistic encoding mechanisms (see [11]), and (b) H.M.’s bilateral lesion *partially* destroyed several hippocampal region structures but *completely* destroyed neither the hippocampus nor any other structure in the region that could in principle house category-specific mechanisms for encoding linguistic and non-linguistic internal representations (see [72]). 

##### 7.2.6.2. English Sentence Plans: How Many Encoding Mechanisms Are Needed?

English has at least eight major lexical categories (nouns, verbs, adjectives, adverbs, pronouns, prepositions, conjunctions, and interjections) plus over 20 lexical subcategories (e.g., common *versus* proper nouns, main verbs *versus* auxiliary verbs) and at least as many phrase (e.g., NPs, VPs) and propositional categories (e.g., subordinate clauses), each with many CCs governing how they combine with other categories (see, e.g., [87,88], for additional categories and CCs). 

If, as discussed in [12], a unique category-specific encoding mechanism is needed to form each unique conjunction of linguistic categories, then hundreds and perhaps thousands of unique encoding mechanisms must underlie the ability to create novel or never previously executed sentence plans in English. To illustrate some of these encoding mechanisms, for a child to create the never previously executed sentence plan “When I visit my doctor in the clinic, she will examine me and help me get better”, one category-specific encoding mechanism is required to conjoin the transitive verb *visit* with the NP *my doctor* to form the VP *visit my doctor*; another is required to conjoin the preposition *in* with the NP *the clinic* to form the prepositional phrase *in the clinic*, another is required to conjoin the main verb *examine* with the auxiliary verb *will* to form the complex verb *will examine*; and another is required to use the coordinating conjunction *and* to conjoin *will examine me* with *help me get better* to form the complex VP *will examine me and* *help me get better*. Given the plethora of category conjunctions that English allows, it thus makes sense that H.M.’s partial hippocampal region damage impaired many but not all of his category-specific mechanisms for encoding novel sentence plans. 

##### 7.2.6.3. Episodic Memories: How Many Encoding Mechanisms Are Needed?

As with English syntax, different types of episodic memories require a plethora of category-specific encoding mechanisms in the hippocampal region. For example, to explain how someone encodes a novel event such as *eating* *dinner at Scalia’s last night*, theories must postulate episodic encoding processes that chunk a unit in the agent category (*I*) with units in (a) the event category (*ate dinner*), (b) the place category (*at Scalia’s*), and (c) the time category (*last night*) to form a new unit in the episodic memory category representing *I ate dinner at Scalia’s last night*.Because there exist many different types of events, places, and times, it therefore makes sense that H.M.’s partial hippocampal region damage impaired many but not all of his category-specific mechanisms for encoding novel, personally experienced events. In any case, without basic categories and category-specific mechanisms for encoding different types of events, places, and times, theories of anterograde amnesia cannot explain spared encoding for specific categories of episodic information, e.g., topics of conversation in case H.M. 

##### 7.2.6.4. Selectively Spared Encoding Categories: Other Sources of Evidence

Other sources of evidence for lesion-specific impairment and sparing of the mechanisms for encoding particular categories of stimuli increase the plausibility of the lesion-specificity account. For example, in short-delay matching-to-sample tasks, hippocampal lesions impair the encoding of location, but spare the encoding of color in passerine birds [89], illustrating category-specific sparing and impairment analogous to H.M.’s spared encoding of the gender, number, and person for proper names but not for other ways of referring to people (see [90], for additional examples of lesion-specific impairment and sparing of encoding categories). 

#### 7.2.7. Why Can H.M. Detect and Correct Proper Name Errors?

Present results directly address a question raised in 1.1: Why did H.M. detect, mark, and correct proper name errors but not other types of self-produced errors in a wide range of linguistic and non-linguistic tasks? The answer is that error detection requires comparison between (a) one’s fully encoded sentence plan or intention, and (b) the output containing the error. Because H.M.’s comparison processes are intact (see [23]) and his mechanisms for encoding proper name plans are intact under the lesion-specificity hypothesis, H.M. can therefore detect his proper names errors by comparing his fully encoded proper name plans with his proper name outputs. H.M. can then signal occurrence of proper name errors via error markers such as “no” or “I mean” because his error marking processes (given error detection) are also intact (see [23]). Finally, after detecting a proper name error, H.M. can correct it simply by activating his original, accurately encoded proper name intention. 

However, H.M cannot detect, mark, and correct a wide range of other types of encoding errors because under the lesion-specificity hypothesis, his sentence plans lack fully encoded pronoun-referent conjunctions, determiner-common noun conjunctions, modifier-common noun conjunctions, verb-modifier conjunctions, auxiliary-main verb conjunctions, verb-object conjunctions, subject-verb conjunctions, propositional conjunctions, and correlative conjunctions (see e.g., [24]). As a consequence, H.M. can’t register the mismatch (between planned *versus* actual output) required to detect, mark, and correct his violations of these CCs during encoding (see [23]). 

### 7.3. Compensation Processes in Amnesia

Present and past results indicate that H.M. developed and used four types of compensation strategies discussed next: proper name compensation strategies; word-, phrase-, and proposition-level compensation strategies; familiarity-based compensation strategies; and repetition-based compensation strategies. 

#### 7.3.1. Proper Name Compensation Strategies

Three sets of results suggest that H.M. used proper names to offset his encoding problems involving pronouns, common nouns, and common noun NPs, the only other ways for referring to people. First, H.M. violated gender, person, and number CCs involving pronoun antecedents, pronoun referents, and common noun referents reliably more often than the controls in Study 2, indicating that compensation was necessary to offset his problems with these standard ways of referring to people. Second, H.M. violated no corresponding CCs involving proper names in Study 2, indicating that he could in principle use proper names to compensate for those problems. Third, H.M. overused proper names relative to controls on the TLC ([2], Study 1) and when answering episodic memory questions ([2]; Study 2), expected outcomes given proper name compensation. 

H.M.’s invented proper names were nevertheless difficult for his listeners to comprehend because he failed to introduce them with prefaces such as *Let’s call him* (or *this man*) *David.* These missing introductory prefaces nonetheless provide another clue to the motivation behind H.M.’s proper name compensation strategy: To produce such prefaces, H.M. would have to use the very categories he was trying to avoid: pronouns (e.g., *him* in *Let’s call him…*) and common noun NPs (e.g., *this man* in *Let’s call this man…*). 

#### 7.3.2. Word- and Phrase-Level Compensation Strategies

Based on three sets of results, H.M. produced word- and phrase-level free associations to compensate for his difficulties with the primary demand characteristics of the TLC: to accurately describe a picture using two or three target words in a single grammatical sentence. First, H.M. produced reliably more word- and phrase-level free associations than controls in Study 1. Second, H.M. could in principle compensate for his new-encoding difficulties via free associative retrieval of familiar phrases using his intact retrieval mechanisms (see Study 2; and [2]). Third, H.M.’s word- and phrase-level free associations benefited his TLC performance either directly, e.g., by increasing target word inclusion, or indirectly, e.g., by rendering his responses more easily understood. Together these results suggest that H.M.’s phrase-level free associations served to compensate for his inability to create phrases and propositions that are novel, coherent, grammatical, and readily understood (see also [5,11,13,22,24,31]), much like his proposition-level free associations in MacKay *et al.* [2].

#### 7.3.3. Familiarity-Based Compensation Strategies

H.M. used familiar clichés (stock or formulaic phrases and propositions) reliably (*p* < 0.001) more often than memory-normal controls in MacKay *et al.* [22]. To illustrate H.M.’s overuse of clichés, he repeated variants of the expression “I thought of” 93 times when describing 32 ambiguous sentences in MacKay *et al.* [22]. Like his overuse of proper names, H.M.’s cliché use suggests a compensation process that relies on intact retrieval mechanisms: By retrieving familiar (but free-associative) phrases and propositions, H.M. could achieve *local* coherence, despite the *globally* incoherent CC violations that accompanied his attempts to create novel phrases, propositions and sentences using his impaired encoding mechanisms. 

#### 7.3.4. Repetition-Based Compensation Strategies

Past and present results indicate that relative to controls, H.M. overused four types of repetition that differed in surface form but reflected attempts to overcome his difficulties in forming novel phrase- and proposition-level internal representations. 

##### 7.3.4.1. Elaborative Repetitions

By hypothesis, H.M. was able to offset his inability to form novel phrase- and proposition-level plans on the TLC (Study 2C) and in conversational discourse [22] by producing a familiar word or phrase and then repeating it with elaboration. This hypothesis and its supporting data call for refinement of the massive repetition principle discussed in 1.1 and [6,7,8,9,10,15,16,17,18]. Under this massive repetition principle, amnesics exhibit a general tendency to repeat and require *massive* repetition to form novel internal representations. However, three Study 2 results undermine these assumptions: (a) H.M. produced no more stutters and unmodified word string repetitions than controls on the TLC, ruling out a general tendency to repeat; (b) he required only one or two elaborative repetitions rather than *massive* repetition to form phrase- and sentence-level plans on the TLC; and (c) his elaborative repetitions didn’t simply repeat; they elaborated. Perhaps truly massive repetition is only necessary when relatively permanent conjunctions are required, as in learning an unfamiliar skill (see [23]), or arbitrary conjunctions between unrelated categories of units are required, as in classical conditioning. However, very few repetitions may suffice in sentence planning because (a) sentence plans are relatively impermanent, built to last no longer than the sentence being produced, and (b) H.M.’s intact syntactic retrieval mechanisms determined what types of units to conjoin. 

##### 7.3.4.2. Stimulus Rephrasing Repetitions

H.M. produced two types of stimulus rephrasing repetitions in [22] when detecting and describing the two meanings of ambiguous sentences such as *The stout major’s wife stayed home* (where either the major is stout or his wife is stout). First, when the experimenter explained a second meaning that H.M. had failed to detect in an ambiguous sentence, H.M. (unlike the controls) often repeated with rephrasing the last few words of her explanation. For example, when describing a second meaning of the ambiguous sentence *Those who play chess as well as Bill came*, the experimenter concluded with the words “as good as Bill is, came”, which H.M. repeated with rephrasing: “as Bill is, they came”. Like elaborative repetitions, such “echoing” with elaboration seems to reflect an attempt to form phrase- and proposition-level internal representations for interpretations that H.M. had failed to discover on his own. 

Second, when describing the two meanings in ambiguous sentences, H.M. repeated the ambiguous words themselves reliably more often than the controls, often repeating them many times within a single response. For example, when attempting to describe the meanings of *Mary and I approved of his cooking*, H.M. repeated the ambiguous words *his cooking* 25 times, without ever discovering or expressing the second meaning (“the fact that he was cooking”). Such multiple repetitions are remarkable because the experimenter repeatedly asked H.M. to avoid repeating words in the stimulus sentences, but like his elaborative repetitions, such repetitions seem to reflect his (sometimes unsuccessful) attempts to create novel phrases, in this case, phrases that integrate a new meaning with his internal representation for the remainder of an ambiguous sentence. Consistent with this hypothesis, H.M. has no difficulty describing the two meanings of ambiguous words (e.g., *tank*) or phrases (*to run out of*) that are presented in isolation rather than in sentences (see [12]). 

##### 7.3.4.3. Other Rephrasing Repetitions 

When detecting ambiguities in [22], H.M. also repeated (with rephrasing) one or more *unambiguous* words in an ambiguous sentence reliably more often than memory-normal controls. For example, in his response to the ambiguous sentence *The stout major’s wife stayed home*, H.M. produced seven repetitions (with rephrasing) of the unambiguous words *stay* and *home*: “She *stayed home*, *she stayed home* or was not moving around ... Then, uh, sort of, or made to, or *to stay* at *home* was *to stay*, not go out, not leave...” (repetitions in italics). Note that H.M.’s “not moving around” accurately defines the isolated infinitive “to stay” but is contextually inappropriate as an interpretation of the entire phrase (*stayed home*). Not unlike his other repetitions with rephrasing, H.M.’s repetitions of unambiguous words seem to reflect attempts to form contextually integrated representations for words that have multiple meanings in isolation, but not in the context of the stimulus sentences (see [12]). 

#### 7.3.5. Amnesia-Linked Compensation: Other Strategies

H.M. and other amnesics have developed several additional strategies for offsetting or coping with their deficits, including *confabulation* [91], *memory displacement* (e.g., describing a personally experienced pre-lesion event as occurring post-lesion), *memory appropriation* (e.g., describing a hearsay event as personally experienced), and *avoidance* (e.g., describing personal memory problems as a way of avoiding requests to remember; see [92]).

Amnesics have also used external reminders to cope with their memory problems (sometimes with the help of researchers and therapists; see [93]), e.g., diaries of future appointments, plans, and events of the day. However, no evidence indicates that H.M. created or used such reminders, and based on the present results and [11,12,13], this may have been because he found diary entries and other self-produced reminders difficult to create and later comprehend. 

Also based on present results, adopting Lashley’s [1] strategy seems warranted to determine whether other amnesics with partial damage to the hippocampal region selectively overuse some categories of units during sentence planning in order to compensate for other categories with impaired encoding mechanisms. Given category overuse, the critical empirical question is: Do these amnesics produce more encoding errors (that violate CCs by omission or commission and are uncorrected despite prompts) than controls, involving not the encoding categories they overuse, but other categories serving the same function? As a caveat, however, other amnesics with partial damage to the hippocampal region cannot be expected to use H.M.’s familiarity- and repetition-based word-, phrase-, and proposition-level compensation strategies for sentence planning as extensively as H.M. After all, no other amnesic will have had 26 years to develop and use the foundations of language prior to their hippocampal region damage, followed by over 50 years for developing and using strategies to compensate for effects of that damage.

## Supplementary Files

Supplementary File 1 Supplementary Materials (PDF, 142 KB)
