# Supplementary material for: Compensating for Language Deficits in Amnesia II: H.M.’s Spared versus Impaired Encoding Categories
Source: Brain Sci. 2013 Mar 27;3(2):415–59. doi: 10.3390/brainsci3020415 (PMC4061850; doi:10.3390/brainsci3020415)
Supplement: Supplementary File 1 — Supplementary Materials (PDF, 142 KB) [file brainsci-03-00415-s001.pdf]

Supplementary Materials

These supplemental materials contain (a) a justification for the statistical conventions adopted in MacKay, Johnson, Fazel, Hadley & James [1], and (b) the database analyzed in MacKay *et al.* [1].

1. Justification of the Statistical Conventions in MacKay *et al.* [1]

1.1. The 4.0 Absolute Difference Convention

Meaningful statistical comparisons by convention require differences in absolute scores between a patient and the mean for the control group must equal or exceed 4.0. This convention is derived by analogy from the sign test, where differences must equal or exceed 0 *vs.* 4 for statistical analysis (see, e.g., [2]).

1.2. The 0.0 Standard Deviation (SD) Convention

When the *SD* for a control group is 0.0, the difference in absolute scores for a patient minus the mean for the control group is 6.0 *SD*s by standard convention. The reason for this convention is that any numerator divided by 0.0 is ∞, yielding an impossibly large estimate for differences of any size. However, 6.0 *SDs* provides a statistically conservative estimate of absolute score differences in the numerator that equal or exceed 4 versus 0 because smaller numerator differences with non-zero *SDs* yield difference estimates larger than 6.0 *SDs*. For example, Study 1 reports a difference of 7 proper nouns used by a patient versus a mean of 0.0 for a control group ($N = 8$; $SD = 0.0$), a 6.0 *SD* difference by convention. However, note that this 6.0 *SD* difference would have increased to 19.4 *SDs* with a weaker hypothetical result, namely that a single control participant produces a single proper noun, yielding $SD = 0.354$ rather than 0.0 for the controls and a difference of 6.875 rather than 7.0 between the patient and the mean for the controls.

1.3. The 2.0 SDs Significance Convention

Differences in scores for a patient versus the mean for a matched control group in excess of 2.0 *SD*s are considered reliable. This convention is statistically conservative because 95% of the values in a normal distribution fall within 2 *SD*s of the mean, yielding $p = 0.025$ for a one-tailed *t*-test.

2. The Database in MacKay *et al.* [1]

This database was a transcript of all within-trial responses of H.M. and the experimenter on the modified version of the Test of Language Competence (TLC) adapted from Wiig & Secord (1988) [3] and administered in MacKay, James & Hadley (2008) [4]. Also included are H.M.'s responses to the practice stimulus and a model (complete and error-free) description of each TLC word-picture stimulus. MacKay *et al*. describe their transcription procedures and protocol in detail, their criteria for defining a "response", and the rationale underlying the stimulus labels shown here, e.g., Set 1 Picture 2. The label "inaudible" indicates that two or more of the three transcribers in MacKay *et al*. were less than 100% sure of their transcription after repeated replay. Also shown in italics for each TLC stimulus is the subset of HM's responses that made up the smaller MacKay *et al*. database.

Practice Example

Set 1 Picture 1: Target Words—*sad*, *moving*.

Model Description: These two people looking at the van are sad to be moving away.

H.M.: Well…moving.

Exp.: Mmm hmm. And what are the objects in the picture?

H.M.: He's taking a trunk into the tru…to the truck.

Exp.: Mmm hmm. What else is in the picture?

H.M.: And people are just moving. She doesn't got any shoes on either.

Exp.: Mmm hmm. Okay.

H.M.: And, probably next door neighbors.

Exp.: Okay. Okay, so I want you to make a sentence up about this picture using these two words.

H.M.: Well…moving away and these people are prob…must be saddened.

Exp.: Okay. So can you make a sentence of…?

H.M.: Mary's sad to see somebody move away.

Two-word Experimental Stimuli

Set 1 Picture 2: Target Words—*fall*, *leg*.

Model Description: This man is telling him not to fall and break his leg.

H.M.: Seeing how somebody's climbing that mountain, they are discussing it themselves cause (inaudible) stuff he should take.

Exp.: Mmm hmm. So just try to make up a sentence using these two words.

H.M.: David wanted him to fall and to see what lady's using to pull himself up besides his hands.

Exp.: So can you make one sentence up? Using both words.

H.M.: Well I see that Dave did (inaudible) past and he's going up fast.

Exp.: So, you just need to make up a sentence using these two words. So make up a sentence using the two words.

H.M.: Um well he's got a pack and so does each one of those.

Exp.: Yeah, I see that. But again you just need to use these two words to make a sentence up.

H.M.: Just to see how he's legs, see…how he's using his legs to bo…climb.

Exp.: I know, but you're ignoring my question aren't you?

H.M.: Well both of them (inaudible)

Exp.: I know but I just want you to say a sentence using these words.

H.M.: Well…how they have to fall, uh climb, easing up…(inaudible)

Exp.: So what are the two words?

H.M.: Fall and leg.

Exp.: So can you make up a sentence about this picture?

H.M.: Jay had to use (inaudible) climb too.

Exp.: So can you use both these words to make the sentence?

H.M.: And if they don't (inaudible) the legs like he does, then they will fall.

Exp.: Okay, so say that again.

H.M.: *If they don't use legs like he does…and his hands, they could fall.*

Set 1 Picture 4: Target Words—*some*, *and*.

Model Description: The man is telling her that he would like some cake and ice cream.

H.M.: (inaudible)

Exp.: So say that again?

H.M.: *I like some her…what she had.*

Set 1 Picture 6: Target Words—*but*, *job*.

Model Description: She is saying that they are doing a good job but it's not done.

H.M.: I… she wants the house painted the same as him and he wants to mow the lawn.

Exp.: But use the two words to make the sentence.

H.M.: *I want that job… and…but she says, he gotta do the other part first.*

Set 1 Picture 8: Target Words—*because*, *hard*.

Model Description: He is telling him not to try that because it's hard.

H.M.: Yes, I…I (inaudible).

Exp.: What are the two words you want to use?

H.M.: I want to exercise like these two are.

Exp.: I know but you need to use the two words on the top.

H.M.: And that's really hard, (inaudible).

Exp.: Yep. So what are the two words you need to use?

H.M.: Because it's too har…because it's too hard to do it.

Exp.: So make one sentence.

H.M.: He's pointing out different ways.

Exp.: So try to make a sentence using the top two words.

H.M.: I don't want to do it the same way as he do because you can't do it that way.

Exp.: Okay. So what are the two words you need to use?

H.M.: Um because it's too…these two are doing different…

Exp.: I know but you need to use the two words at the top of the page to make one sentence.

H.M.: Yeah, I see (?).

Exp.: Yeah. So you didn't use both words.

H.M.: *Because it's too hard to do it that way.*

Exp.: Okay.

H.M.: (inaudible)

Set 1 Picture 10: Target Words—*first*, *across*.

Model Description: The man is telling the boy to wait first before going across the street.

H.M.: *He wants to cross here…first.*

Set 2 Picture 3: Target Words—*sit*, *painted*.

Model Description: He is telling her not to sit there because it's just been painted.

H.M.: *And that man is trying to tell that woman not to sit there because it's wet paint.*

Exp.: Good.

H.M.: *He can uh see the sign better than she could and she's ready to sit down there.*

Set 2 Picture 5: Target Words—*pie*, *either*.

Model Description: He is telling her that he wants either pie or cake.

H.M.: Since they've got their coffee already he isn't- they just want their uh pie and the piece of this pie up here because the cake is down here.

Exp.: OK, you didn't use this one. What's this word?

H.M.: Pie.

Exp.: No, this one over here.

H.M.: Either.

Exp.: So how would you use that one in the same sentence with pie to describe what is going on there?

H.M.: He hadn't got any milk there or put it in his cup.

Exp.: Do you know what the word either means?

H.M.: Or.

Exp.: OK. (pause) Can you think of one sentence using both of those two words?

H.M.: *Well this pie is—or the pie here was back here—*

Exp.: Uh-huh.

H.M.: *and uh coffee is in there because heat a solid and this is only boiled milk say milk there and this is not liquid but only ice.*

Set 2 Picture 7: Target Words—*crowded*, *drive*.

Model Description: She is telling him that the school bus is so crowded, they should drive.

H.M.: A driving wanna drive some place and this bus is stopped up there.

Exp.: What is this word.

H.M.: *Is it crowded and it just pointed out this bus is up here and it's crowded school bus.*

Set 2 Picture 9: Target Words—*although*, *wrong*.

Model Description: She is saying he should take that suit although it looks wrong on him.

H.M.: Well she's choosing the soup here-

Exp.: Um-hum.

H.M.: for him.

Exp.: OK. What about the words although and wrong? Can you use those words?

H.M.: Yes. *Because it's wrong for her to be and he's dressed just as this that he's dressed and the same way—*

Exp.: OK, good.

H.M.: *as her.*

Set 2 Picture 11: Target Words—*fresh*, *nor.*

Model Description: She is telling her that the bread looks neither fresh nor healthy.

H.M.: *Well you—she wants one thing and he wants another thing and the fresh are not—are not. Doesn't say that, it says not.*

Exp.: It says nor.

H.M.: She doesn't want her pie.

Exp.: It says nor. Do you know the word nor?

H.M.: Yeah. Or she could say this. This is in (inaudible) over here and this is just little things (inaudible) a little spice you could call eclairs and stuff like that it's over here.

Three-word Experimental Stimuli

Set 1 Picture 3: Target Words—*sit*, *painted*, *because*.

Model Description: He is telling her not to sit on that bench because it was just painted.

H.M.: *Oh, don't sit because it's just been painted.*

Set 1 Picture 5 Target Words—*pie*, *either*, *have*.

Model Description: The man is saying he'll have either the pie or the cake.

H.M.: I want some of that pie either some pie and I'll have some.

Exp.: So say that again.

H.M.: I'll have some of that pie and that she's having.

Exp.: So you, but you need to use these three words.

H.M.: Well pie, either, and.

Exp.: So put those into one sentence.

H.M.: I'll have pee with pie or (inaudible) hers and (inaudible). Cause there's a cake down here.

Exp.: Yeah. So you still haven't used all those words in one sentence.

H.M.: There's one kind of pie and there's another kind of pie.

Exp.: Okay. So can you put them into a sentence.

H.M.: And he wants the same par…kind that she does.

Exp.: But you're not using these three words.

H.M.: Well, they both have to use pie.

Exp.: I know, but you have to use the other two words as well.

H.M.: Any pie to either have.

Exp.: What was that?

H.M.: *Any pie that either she either had.*

Set 1 Picture 7: Target Words—*crowded*, *drive*, *if*.

Model Description: The woman is saying that he can drive that crowded bus if he wants to.

H.M.: *Melanie tra…on that bus, the scrawny bus and have it drive it off…it, it drives it off.*

Exp.: So say that again.

H.M.: Melanie gets on that one if she can and she wants her to travel along with him.

Exp.: Okay. So try to use the three words at the top to make one sentence.

H.M.: Well he has to go the same way as her if (inaudible)…she wants to go on the bus…and it's crowded…it's crowded.

Exp.: Okay.

H.M.: Too crowded to get on the bus.

Exp.: Okay.

H.M.: (inaudible)…one way out, it's on common street.

Set 1 Picture 9: Target Words—*actually*, *although*, *wrong*.

Model Description: She is saying she actually likes that suit although it is wrong for him.

H.M.: He had this (inaudible)…no, she's taking that suit and he wants to take it…and he's trying to sell.

Exp.: So how can you use the top three words to make a sentence?

H.M.: Actually…he's in this (inaudible) pointing (inaudible) dresses over here…he wants…he wants this kind of color too. And she wants something similar to that.

Exp.: Okay. So make a sentence using the top three wor…top words.

H.M.: *Actually it's best for him. It's wrong for her. They have 'em the same way.*

Set 1 Picture 11: Target Words—*fresh*, *nor*, *here*.

Model Description: The pie here looks neither fresh nor good.

H.M.: Once has to be trash in yellow (inaudible)…is not here.

Exp.: So can you say that again?

H.M.: So, this is (inaudible) *Gary is…almos…almost…hasn't been cut the same way. And his (inaudible)*

just what they are there.

Exp.: Okay. So can you make a sentence up?

H.M.: (inaudible)…here.

Set 2 Picture 2: Target Words—*fall*, *leg*, *and*.

Model Description: This man is telling him not to fall and break his leg.

H.M.: Fall, leg, T and uh, and.

Exp.: OK, good.

H.M.: I should say fall, leg, and.

Exp.: There ya go. So, now can you make up a sentence that has the words fall, leg, and and in it that describes what is going on in this picture?

H.M.: He's climbing that and he can fall.

Exp.: OK, so that has two of the words. You've used and and fall. You said, he's climbing that and he can fall.

H.M.: *And he has to use his legs to call-climb*.

Set 2 Picture 4: Target Words—*some*, *and*, *get.*

Model Description: He is telling her he wants to get some cake and pie.

H.M.: Well he's putting the price of it and price of thing what it is and she wants to (inaudible) in there and he's waitin' to be waited on.

Exp.: OK, but you didn't use these three words here.

H.M.: *He is getting some of this and it isn't pointed out here what it is and he is just waiting to get waited on.*

Set 2 Picture 6: Target Words—*job*, *but*, *easy.*

Model Description: She is saying it won't be easy but they need to do a good job.

H.M.: Job, but, easy.

Exp.: OK, so can you think of a sentence that uses all three of those words that describes that picture?

H.M.: *It is easy to paint the place even though it's been just a job and easy on the job part*.

Set 2 Picture 8: Target Words—*because*, *hard*, *like.*

Model Description: He is saying he doesn't like to do that because it is so hard.

H.M.: *"Cause he's doin" that and this one liked to do it this way to sit down*.

Exp.: Um-hum.

H.M.: *And this could be hard here and soft here*.

Exp.: Good. So the floor is hard and the trampoline's soft huh?

H.M.: Yeah.

Set 2 Picture 10: Target Words—*before*, *first*, *across.*

Model Description: The father is telling his son to look first before going across the street.

H.M.: Before at first you cross across.

Exp.: OK, good. How would you use those three words to describe that picture?

H.M.: *Before you cross the street you have to look both ways first*.

References

1. MacKay, D.G.; Johnson, L.W.; Fazel, V.; James, L.E. Compensating for language deficits in amnesia I: H.M.'s spared retrieval categories. *Brain Sci.* **2013**, *3* (1), 262–293; doi:10.3390/brainsci3010262.

2. Siegel, S. Castellan, N.J. *Nonparametric Statistics for the Behavioral Sciences.* McGraw-Hill: New York, NY, USA, 1956.
3. Wiig, E.H.; Secord, W. *Test of Language Competence: Expanded Edition*. Pearson Assessments: New York, NY, USA, 1988.
4. MacKay, D.G.; James, L.E.; Hadley, C. Amnesic H.M., language production, and memory: Selective deficits on the Test of Language Competence. *J. Exp. Clin. Neuropsychol.* **2008**, *30*, 280–300.
